# Sialic acid exacerbates gut dysbiosis-associated mastitis through the microbiota-gut-mammary axis by fueling gut microbiota disruption

**DOI:** 10.1186/s40168-023-01528-8

**Published:** 2023-04-17

**Authors:** Caijun Zhao, Xiaoyu Hu, Min Qiu, Lijuan Bao, Keyi Wu, Xiangyue Meng, Yihong Zhao, Lianjun Feng, Shiyu Duan, Yuhong He, Naisheng Zhang, Yunhe Fu

**Affiliations:** 1 Department of Clinical Veterinary Medicine, College of Veterinary Medicine, Jilin University, Changchun, 130062 Jilin Province China; 2 Department of Breast Center, West China Hospital, Sichuan University, Chengdu, 610041 Sichuan Province China

**Keywords:** Sialic acid, Mastitis, Gut microbiota, Microbiota-gut-mammary axis, *Enterobacteriaceae*, *Moraxellaceae*

## Abstract

**Background:**

Mastitis is one of the most severe diseases in humans and animals, especially on dairy farms. Mounting evidence indicates that gastrointestinal dysbiosis caused by induction of subacute ruminal acidosis (SARA) by high-grain diet consumption and low in dietary fiber is associated with mastitis initiation and development, however, the underlying mechanism remains unknown.

**Results:**

In the present study, we found that cows with SARA-associated mastitis have altered metabolic profiles in the rumen, with increased sialic acids level in particular. Consumption of sialic acid (SA) in antibiotic-treated mice, but not healthy mice, induced marked mastitis. SA treatment of antibiotic-treated mice also induced mucosal and systemic inflammatory responses, as evidenced by increased colon and liver injuries and several inflammatory markers. In addition, gut dysbiosis caused by antibiotic impaired gut barrier integrity, which was aggravated by SA treatment. SA potentiated serum LPS level caused by antibiotic treatment, leading to increased activation of the TLR4-NF-κB/NLRP3 pathways in the mammary gland and colon. Moreover, SA facilitated gut dysbiosis caused by antibiotic, and especially enhanced *Enterobacteriaceae* and *Akkermansiaceae*, which correlated with mastitis parameters. Fecal microbiota transplantation from SA-antibiotic-treated mice mimicked mastitis in recipient mice. In vitro experiments showed that SA prompted *Escherichia coli* growth and virulence gene expression, leading to higher proinflammatory cytokine production in macrophages. Targeting the inhibition of *Enterobacteriaceae* by sodium tungstate or treating with the commensal *Lactobacillus reuteri* alleviated SA-facilitated mastitis. In addition, SARA cows had distinct ruminal microbial structure by the enrichment of SA-utilizing opportunistic pathogenic *Moraxellaceae* and the depletion of SA-utilizing commensal *Prevotellaceae*. Treating mice with the specific sialidase inhibitor zanamivir reduced SA production and *Moraxellaceae* abundance, and improved mastitis in mice caused by ruminal microbiota transplantation from cows with SARA-associated mastitis.

**Conclusions:**

This study, for the first time, indicates that SA aggravates gut dysbiosis-induced mastitis by promoting gut microbiota disturbance and is regulated by commensal bacteria, indicating the important role of the microbiota-gut-mammary axis in mastitis pathogenesis and suggesting a potential strategy for mastitis intervention based on gut metabolism regulation.

Video Abstract

**Supplementary Information:**

The online version contains supplementary material available at 10.1186/s40168-023-01528-8.

## Introduction

The complex intestinal mutualistic community, referred to as the gut microbiota, has been proven to be one of the most important regulators affecting physiological homeostasis and disease outcomes [1, 2]. A large number of studies indicated that the gut microbiota regulates host health through nutritional absorption, barrier maintenance, immune and metabolic regulation [3], and disruption of the fragile balance in the gut microbiota, termed gut dysbiosis [4], plays a central role in the initiation, development, and outcome of many diseases, including inflammatory bowel disease [5], fatty liver [6], obesity [7], cancer [4], and infections [8]. Commonly, metabolic alterations derived from gut microbiota are the most direct way of mediating host-microbiota interactions and influencing disease progression. For example, gut microbiota-derived short-chain fatty acids (SCFAs) contribute to maintaining intestinal anaerobic conditions [9], regulating mucosal and systemic Treg/Th17 balance [10], and are involved in the metabolic fitness of the brain innate immune system [11]. Microbiota-catabolized ligands of the aryl hydrocarbon receptor (AhR) from dietary tryptophan reduce central nervous system inflammation through regulating astrocyte activity by type I interferons and microglial-astrocyte interaction [12, 13]. On the other hand, gut dysbiosis-derived metabolites increase the risk for diseases. The microbial metabolite 4-ethylphenyl sulfate (4-EPS) can alter brain activity and anxiety behavior by impairing oligodendrocyte maturation and reducing oligodendrocyte-neuron interactions [14]. Koh et al. found that histidine-derived imidazole propionate by the gut microbiota impairs glucose tolerance and insulin signaling through p38-mediated activation of the mechanistic target of rapamycin complex 1 [15]. Moreover, gut microbiota production of trimethyl-5-aminovaleric acid (TMAVA) facilitates cardiac hypertrophy by reducing fatty acid oxidation [16]. These results suggest that exploring host or microbiota-mediated metabolic alterations is important for achieving an in-depth understanding of the pathogenesis of gut microbiota-associated distal diseases.

Mastitis is a common and severe disease for humans and animals that increases the risk for breast cancer [17, 18] and impairs milk yield and quality, making it a major threat to women's health and hindering the development of the dairy industry. Mounting evidence suggests a possible “microbiota-gut-mammary axis” in the context of mastitis as shown by gut dysbiosis initiating and facilitating the development of mastitis. In detail, studies reported that fecal microbiota transplantation (FMT) from mastitis cows to mice induces mastitis [19, 20]. Our previous studies also demonstrated that antibiotic-treated mice have increased susceptibility to *Staphylococcus aureus* (*S. aureus*)- and *Escherichia coli* (*E. coli*)-induced mastitis [21, 22], confirming the important role of the microbiota-gut-mammary axis in mastitis pathogenesis. A previous study showed that alteration of the gut microbiota in response to different dietary patterns induced distinct mammary metabolic profiles [23]. Impaired beneficial metabolite levels, including SCFAs and microbiota-metabolic AhR ligands, are reported to regulate the outcome of pathogen-induced mastitis in mice [21, 22]. However, little is known about the direct link between gut microbial metabolites and the risk of mastitis.

There is an increased feed ratio in a high-grain diet (HGD) due to the high-nutritional demand of cows during the milk period and the pursuit of milk yield and quality in dairy farms. However, excessive consumption of a HGD and low in dietary fiber will reduce the ruminal pH and disrupt the composition and functions of the ruminal microbiota, a phenomenon referred to as subacute ruminal acidosis (SARA) [24]. A previous study found that SARA cows have increased susceptibility to pathogen-induced mastitis [25]. Our previous result also supported the hypothesis that SARA may serve as a driver for mastitis, as shown by the long term consumption of a HGD increasing systemic inflammatory responses and mastitis [26, 27], which was associated with increased ruminal lipopolysaccharide (LPS) levels derived from the enriched Gram-negative bacteria in SARA cows [26, 28]. However, whether dysbiosis-induced metabolic changes promote the expansion of ruminal opportunistic pathogen and LPS release and regulate the development of mastitis is poorly understood.

In the present study, we investigated the ruminal metabolic and microbial profile of cows with SARA by using untargeted metabolomics and 16S rRNA sequencing. Based on metabolomics results and correlation analysis, we focused on the role of sialic acids (SAs, N-glycolylneuraminic acid (Neu5Gc) and N-acetylneuraminic acid (Neu5Ac)) in mastitis pathogenesis and the potential mechanism by using antibiotic-treated mouse model. Our results demonstrated that SA exacerbates gut dysbiosis-induced mastitis by fueling microbiota disruption through the microbiota-gut-mammary axis, which provides insight into the pathogenesis of mastitis and acts as the basis for finding potential strategies for mastitis intervention.

## Results

### Establishment of a cow SARA-associated mastitis model and experimental design

**Fig. 1:**
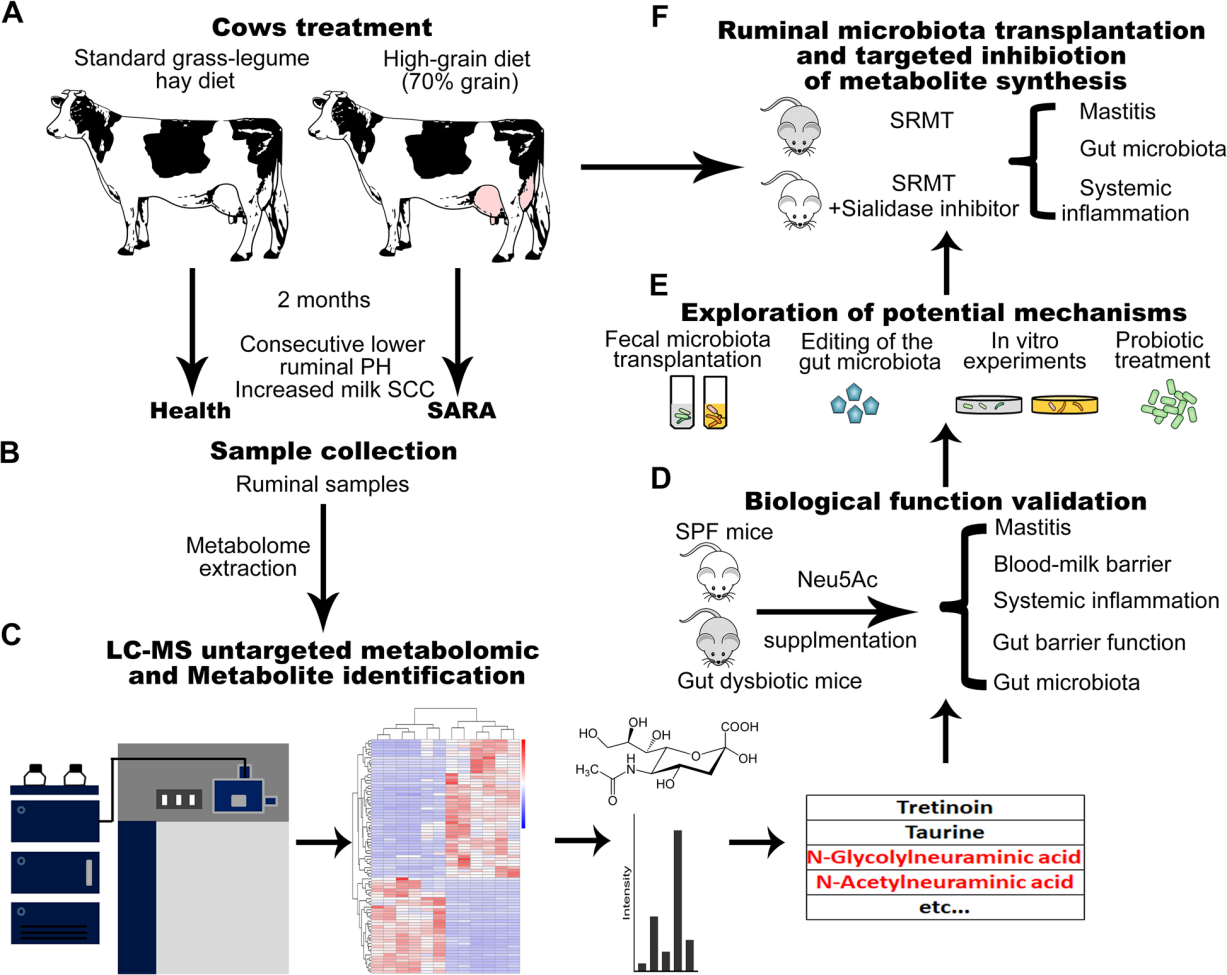
Experimental design Cows were treated with a standard or high-grain diet for 8 weeks (**A**) and ruminal samples were collected for metabolomic analysis (**B**). **C** Untargeted metabolomics was performed by LC–MS and metabolites that were significantly different between healthy and SARA cows were identified. **D** The biological functions of sialic acid were validated in mice. **E** The underlying mechanism of potential metabolites on mastitis pathogenesis was investigated by fecal microbiota transplantation, precision editing of gut microbiota, in vitro experiments and probiotic treatment. **F** Ruminal microbiota transplantation and pharmacological sialidase inhibition were performed to confirm the role of sialic acid. LC–MS, liquid chromatography-mass spectrometry; Neu5Ac, N-acetylneuraminic acid; SARA, subacute ruminal acidosis; SCC, somatic cell count; SPF, specific pathogen free; SRMT, SARA ruminal microbiota transplantation

To investigate the relevance between SARA and mastitis in cows, we first established cow SARA model by treating cows with a HGD (70% grain and 30% forage) for 8 weeks as previously described [27, 29]. The control cows were fed with a standard grass-legume hay diet (Fig. 1A). SARA was diagnosed by a ruminal pH persistently below 5.8 as previously described (data not shown) [27]. After 8 weeks of consumption of a HGD, the pH values of the SARA cows were significantly lower than those of healthy cows (Figure S1A). We then found that SARA cows had increased ruminal and serum LPS levels and increased somatic cell cunt (SCC) in the milk (Figure S1B–D). SCC was commonly used in the diagnosis of cow mastitis on dairy farms [30, 31]. To confirm the mastitis, we performed mammary gland histological analysis and found that SARA cows had increased inflammatory changes, as shown by edema, inflammatory exudation and granulocyte infiltration (Figure S1E–F). Consistent with these results, we showed that SARA cows had increased proinflammatory cytokines TNF-α and IL-1β in the mammary gland (Fig. S1G, H). These results indicated that SARA can induce mastitis symptoms in dairy cows.

To investigate the potential metabolites involved in the pathogenesis of SARA-mediated mastitis, untargeted metabolomics profiling was performed on 12 ruminal samples from SARA (*n* = 6) and healthy (*n* = 6) cows using high-performance LC–MS (Fig. 1B). Integrative analysis was performed based on altered metabolites in the rumen and correction with mastitis indices (Fig. 1C). The biological function of the candidate metabolite was validated in specific-pathogen free (SPF) mice and antibiotic-treated mice (Fig. 1D). Moreover, the underlying mechanism of potential metabolite on mastitis pathogenesis was investigated by FMT, precision editing of gut microbiota, probiotic treatment and in vitro experiments (Fig. 1E). Finally, we confirmed the potential role of candidate metabolite in mastitis pathogenesis by performing ruminal microbiota transplantation (RMT) from cow to mouse and treating mice with targeted inhibitor simultaneously (Fig. 1F).

### SARA cows have altered metabolite levels in the rumen

**Fig. 2:**
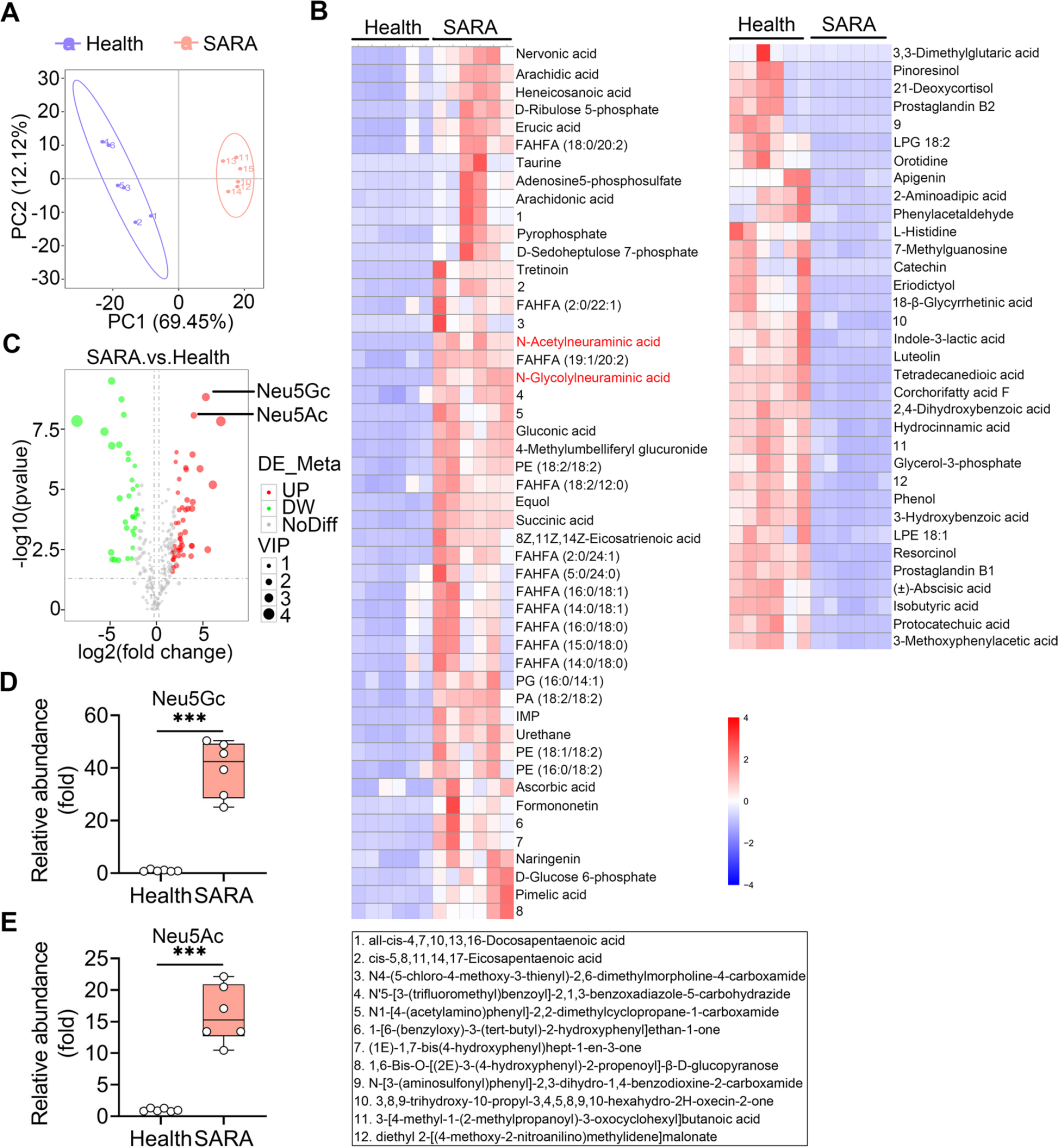
Metabolic profiles of health and SARA cows.** A** PCA score plots for ruminal samples. The *X* axis and *Y* axis represent the percentages of contribution the first two principal components (PC1 and PC2) (*n* = 6). **B** Hierarchical cluster analysis of ruminal metabolites that were different between health and SARA cows (*n* = 6). **C** Volcano plots indicating the results of pairwise comparisons of metabolites in health and SARA cows (*n* = 6). The vertical and horizontal dashed lines indicate the threshold for the twofold abundance difference and the *p* = 0.05 threshold, respectively. Student’s *t* test was performed for the comparison. Significant metabolites are presented in red (upregulated) or green (downregulated) (VIP > 1, *P* value < 0.05 and FC ≥ 2 or FC ≤ 0.5). The relative expression levels of Neu5Gc (**D**) and Neu5Ac (**E**) in health and SARA samples were determined (*n* = 6). Each dot represents an individual and Student’s *t* test was performed, followed by Tukey test. ***p < 0.001. Neu5Gc, N-Glycolylneuraminic acid; VIP, variable importance in the projection

To acquire stable and accurate metabolome results, data quality control (QC) was performed through Person correction analysis. The Pearson correlation of ruminal QC samples was high, suggesting reliable data quality (Figure S2A). Moreover, the QC samples clustered tightly together in the principal component analysis (PCA) score plots (Figure S2B, C), which further confirmed the reliable data quality. A total of 282 annotated metabolites were identified in all ruminal samples by using the Human Metabolome Database (HMDB), Kyoto Encyclopedia of Genes and Genomes (KEGG) and LIPID MAPS (Figure S3A–C, Table S1). PCA showed that SARA ruminal samples were significantly separated from healthy ruminal samples (PC1 was 69.45% and PC2 was 12.12%, Fig. 2A). Moreover, distinct clusters of SARA cows compared with healthy cows were confirmed by partial least squares discrimination analysis (PLS-DA) score plots (*p* < 0.001, Figure S4A). The stability and reliability of PLS-DA were confirmed by sevenfold cross validation (R2Y:0.99, Q2Y:0.98, Figure S4A). Furthermore, a model containing metabolic information by fitting PLS-DA through a random permutation testing 200 times was performed to confirm the ability of PLS-DA to correctly classify new samples. The results demonstrated that the model is reliable and does not overfitting, as shown by the intercepts of goodness-of-fit (R2) being greater than goodness-of-prediction (Q2) and the intercept of Q2 being less than zero (Figure S4B).

We identified 83 significantly differential metabolites in SARA cows compared with healthy cows (49 upregulated and 34 downregulated, Table S1, S2, and S3) by considering variable importance in the projection (VIP), fold change (FC) and P value (VIP > 1, FC ≥ 2, or FC ≤ 0.5 and *P* value < 0.05). Moreover, the metabolite levels in SARA cow rumens were significantly different from those in healthy cows through differential metabolite cluster analysis by using a heatmap (Fig. 2B). KEGG pathway enrichment analysis indicated that pathways enriched in SARA cows were mainly protein digestion and absorption, serotonergic synapse, pentose phosphate pathway and arachidonic acid metabolism (Figure S4C). The global distribution of different metabolites was shown by volcano plots, which indicated distinct metabolite levels between SARA and healthy ruminal samples (Fig. 2C). We further performed correlation analysis between significant metabolites and clinical inflammatory markers. The results showed that 22 metabolites were positively correlated with inflammatory markers, while 8 metabolites were negatively correlated with inflammatory markers (Figure S5). A total of 6 metabolites were upregulated in SARA cows with more than 16-fold FC (Fig. 2C), and 5 of them were positively correlated with inflammatory markers (Figure S5), including tretinoin, formononetin, Neu5Gc, 8Z,11Z,14Z-eicosatrienoic acid, and Neu5Ac. Among these, Neu5Gc and Neu5Ac had higher P values by a -log10 *p* value of more than 8 (Fig. 2C, Table S2). Collectively, these results indicated that SARA cows have altered metabolite levels, some of which were correlated with SARA-associated mastitis parameters.

### Consumption of SA exacerbates gut dysbiosis-induced mastitis in mice

Neu5Gc and Neu5Ac are nine-carbon backbone monosaccharides that are commonly known as SAs, and are abundantly displayed on all mucosal surfaces [32]. Neu5Gc is the modified form of Neu5Ac produced by cytidine monophosphate N-acetylneuraminic acid hydroxylase [32]. Levels of SA are very low in the gastrointestinal tract under normal conditions, while adverse factors, such as gut dysbiosis or mucosal inflammation, can increase free SA levels by increasing the expression of bacterial sialidases or host sialyltransferase, respectively [33, 34]. We showed a 39.7-fold FC and 16.1-fold FC increase in Neu5Gc and Neu5Ac in SARA cows respectively (Fig. 2D, E, Table S2). Different metabolite correlation analyses also found that Neu5Gc and Neu5Ac were positively correlated with tretinoin (Figure S6), the metabolite with the greatest increase in SARA cows according to the FC that has been reported at inflammation sites and occurs concurrently with the development of inflammation [35]. We next focused on the potential effects of SA on SARA-associated mastitis pathogenesis.

**Fig. 3:**
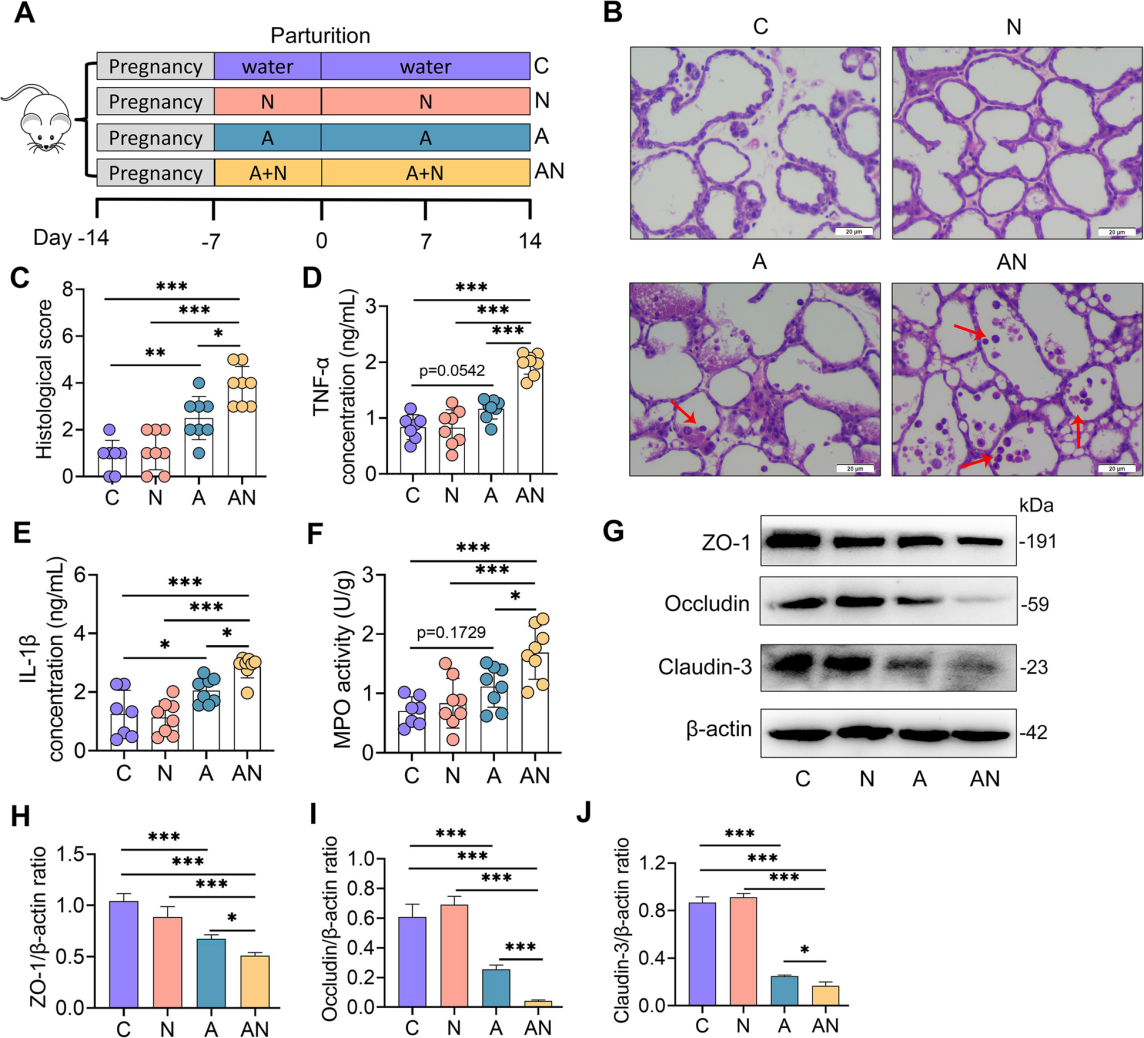
Sialic acid exacerbates gut dysbiosis-induced mastitis in mice. **A** Experimental protocol of the sialic acid treatment. Mice were treated with free water, Neu5Ac, ampicillin or ampicillin + Neu5Ac for the indicated times. The mammary tissues were harvested 14 days after parturition. **B** Histological analysis of mammary tissues using H&E-stained sections (scale bar, 20 μm). Red arrows indicate leukocyte infiltration. **C** Histological scores of mammary gland in different groups based on H&E-stained sections (*n* = 7–8). Inflammatory parameters of mammary glands from different groups, including TNF-α (**D**) and IL-1β (**E**) concentrations and MPO activity (**F**), were measured (*n* = 7–8). Representative protein levels of ZO-1, Occludin and Claudin-3 (**G**) and intensity analysis of ZO-1, Occludin, and Claudin-3 from different treated mice (**H-J**) were determined (*n* = 4). Data are expressed as the mean ± SD (**C–F** and **H–J**). One-way ANOVA was performed, followed by Tukey test (**C–F** and **H–J**). *p < 0.05, **p < 0.01, ***p < 0.001 indicate significant difference. A, ampicillin; C, control; N, Neu5Ac; AN, ampicillin + Neu5Ac

To investigate the role of SA in the pathogenesis of mastitis, we supplemented mice with Neu5Ac (hereafter referred to as SA) in normal or antibiotic-induced disturbance of gut microbiota according to previous studies [21, 22, 36] (Fig. 3A), which demonstrated that long-term gut dysbiosis initiated mastitis in mice. We first identified that treatment with SA increased intestinal SA levels in both conventional and antibiotic-treated mice (Figure S7A). Interestingly, gut-dysbiotc mice also showed mild increase in SA compared with control group (Figure S7A). The histological score of the mammary gland showed that SA treatment (N group) had no significant influence on mammary tissues (Fig. 3B, C). Antibiotic-treated mice (A group) had little leucocyte infiltration, while antibiotic-treated mice supplemented with SA (AN group) exhibited significant mammary leucocyte infiltration (Fig. 3B, C). Consistently, the N group had no significant changes in tumor necrosis factor (TNF)-α, interleukin (IL)-1β and myeloperoxidase (MPO) levels (Fig. 3D–F) compared with the C group, while the AN group had increased proinflammatory cytokine levels compared with the A group (Fig. 3D–F). However, we observed minimum effects of SA treatment on milk production when assessing the gene expression associated with milk protein synthesis including *Csn1-3* and *Wap* (Figure S8A–D), or on the weight gain of pups (Figure S8E). We further investigated the effect of SA on the blood-milk barrier by assessing the expression of the tight junction (TJ) protein ZO-1, Occludin, and Claudin-3, which are disrupted during mastitis [21, 22, 37]. The results demonstrated that SA treatment did not reduce TJ protein levels in the mammary gland compared with control treatment (Fig. 3G–J). However, the A group had reduced ZO-1, Occludin and Claudin-3 compared with the C group (Fig. 3G–J), and these decreases were aggravated in the AN group (Fig. 3G–J). These results indicated that SA contributes to the development of mastitis in antibiotic-treated mice.

### SA promotes mucosal and systemic immune imbalance in antibiotic-treated mice

**Fig. 4:**
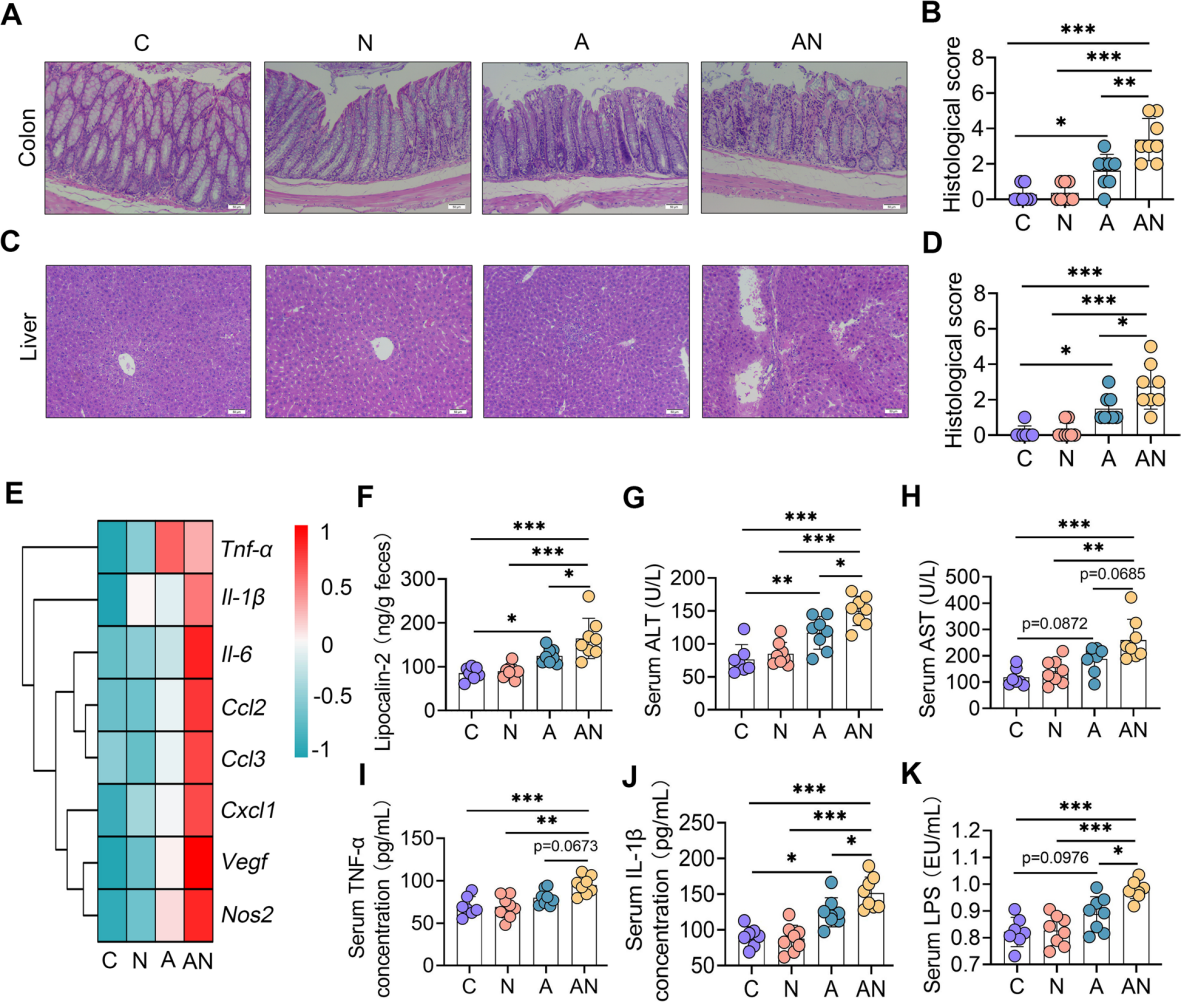
Sialic acid facilitates gut dysbiosis-induced mucosal and systemic immune imbalance in mice.** A** Representative H&E-stained colon sections from the indicated mice (scale bar, 50 μm). **B** Histological score of colons from different treatment groups (*n* = 7**–**8). **C** Representative H&E-stained liver sections from different groups (scale bar, 50 μm). **D** Histological scores of the livers based on H&E staining (*n* = 7**–**8). **E** Colon mRNA levels from the indicated mice as assessed by qPCR (*n* = 7**–**8). **F** Fecal lipocalin-2 levels were assessed to validate mucosal inflammatory responses (*n* = 7**–**8). Serum ALT (**G**) and AST (**H**) levels were determined to assess liver injury (*n* = 7**–**8). Levels of serum TNF-α (**I**), IL-1β (**J**), and LPS (**K**) showed the systemic inflammatory responses caused by gut dysbiosis and sialic acid treatment (*n* = 7**–**8). Data are expressed as the mean ± SD (**B**,** D**, and **F–K**) and one-way ANOVA was performed, followed by Tukey test (**B, D,** and **F–K**). *p < 0.05, **p < 0.01, ***p < 0.001 indicate significant difference

We further studied the effects of SA on systemic inflammation. Histological analyses of the ileum, colon and liver were performed, and the results showed that the N group had no changes in the ileum, colon or liver compared with the C group (Fig. 4A–D and Figure S8F, G). However, the A group demonstrated inflammatory changes in the colon and liver, and these changes were exacerbated in the AN group (Fig. 4A–D). No significant changes were observed in the ileum among the different treatments (Figure S8F, G). Moreover, we found that the N group had the same levels of proinflammatory genes in the colon as the C group by qPCR (Fig. 4E), but the A group had increased proinflammatory gene expression and these increases were aggravated in the AN group (Fig. 4E). Consistently, the A group had increased lipocalin-2 levels compared with the C group, a marker of mucosal inflammation [38], and the AN group had higher lipocalin-2 levels in the feces than the A group (Fig. 4F). Furthermore, we observed increased serum alanine aminotransferase (ALT) and aspartate aminotransferase (AST) levels in the A group compared with the C group, while these increase were exacerbated in the AN group (Fig. 4G, H). In addition, we showed that the A group had higher serum TNF-α, IL-1β, and LPS levels than the C group (Fig. 4I–K), and these increases were enhanced in the AN group (Fig. 4I–K). Of note, there were no significant differences in these inflammatory markers between the C and N groups (Fig. 4F–K). Taken together, these results indicated that SA enhances mucosal and systemic inflammatory responses caused by antibiotic-induced gut dysbiosis.

### SA aggravates gut barrier disruption in antibiotic-treated mice

**Fig. 5:**
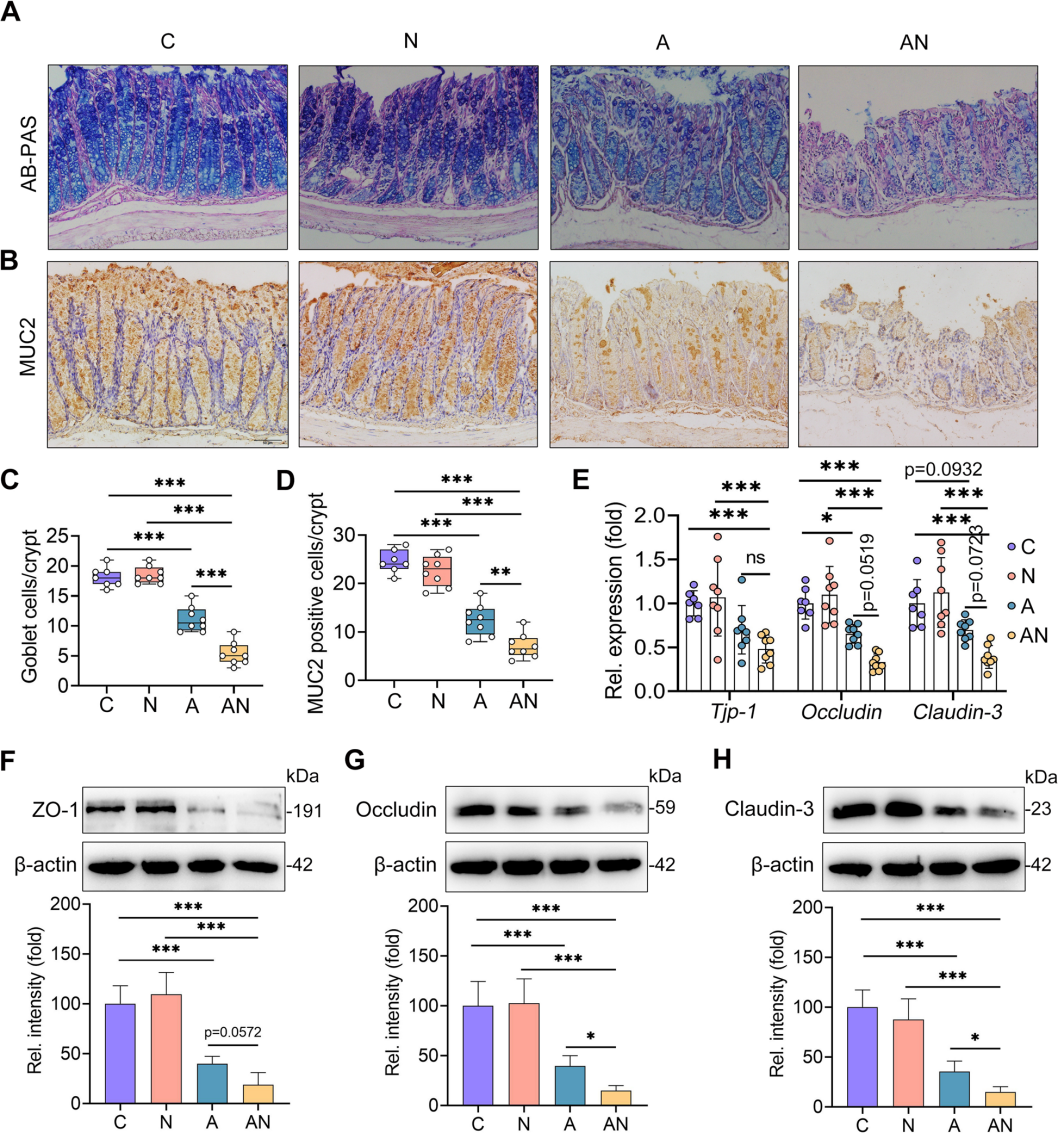
Sialic acid promotes gut dysbiosis-induced intestinal barrier disruption. The goblet cells of colons from different treated mice are shown by AB-PAS staining (scale bar, 50 μm) (**A**, **C**) (*n* = 7**–**8). Immunohistochemistry staining was performed to assess the mucin-2 level in the colon using mucin-2 antibody (scale bar, 50 μm). The positive-stained cells are shown in brown (**B**, **D**) (*n* = 7**–**8). **E** Relative mRNA levels of colon tight junction proteins, including ZO-1, Occludin, and Claudin-3, from the indicated mice were assessed by qPCR (*n* = 7**–**8). Protein levels of colon ZO-1 (**F**), Occludin (**G**), and Claudin-3 (**H**) were measured by western blotting (*n* = 4). Data are expressed as the mean ± SD (**C**–**H**) and one-way ANOVA was performed, followed by Tukey test (**C**–**H**). *p < 0.05, **p < 0.01, ***p < 0.001 indicate significance. AB-PAS, Alcian Blue Periodic acid Schiff; MUC2, mucin-2

Mounting evidence suggests that the increased gut barrier permeability is attributed to systemic inflammatory responses in response to gut dysbiosis or inflammation by allowing harmful metabolites or bacterial components to migrate from the gut into the blood [39]. We further showed that the A group had fewer goblet cells in the colon than the C group by alcian blue periodic acid Schiff (AB-PAS) staining (Fig. 5A, C). In the AN group, the gut dysbiosis-induced decrease in goblet cells was aggravated (Fig. 5A, C). Goblet cells contribute to the production of mucin-2, one of the most important mucosal proteins that construct the first line of defense in protecting the intestinal epithelium from intestinal symbiotic bacteria or pathogens [40]. Indeed, we found that the A group had lower mucin-2 levels than the C group (Fig. 5B, D), and this decrease was amplified in the AN group (Fig. 5B, D). Moreover, we assessed the mRNA expression of TJ proteins, including *Tjp-1*, *Occludin*, and *Claudin-3*, in the colon. The results showed that the A group had decreased *Tjp-1*, *Occludin*, and *Claudin-3* mRNA levels compared with the C group, while the AN group had lower mRNA expression of TJPs than the A group, although no significant differences were detected (Fig. 5E). Furthermore, we revealed that antibiotic treatment reduced the protein levels of ZO-1, Occludin, and Claudin-3 in the colon compared with control mice (Fig. 5F–H), and that the decreases in the AN group were aggravated compared with the A group (Fig. 5F–H). Interestingly, we did not observe a significant difference in gut barrier function between the C and N groups (Fig. 5A–H). Collectively, these results indicate that SA aggravates antibiotic-induced intestinal barrier dysfunction but does not affect normal gut barrier function.

### SA potentiates inflammation through the TLR4-NF-κB/NLRP3 signatures

**Fig. 6:**
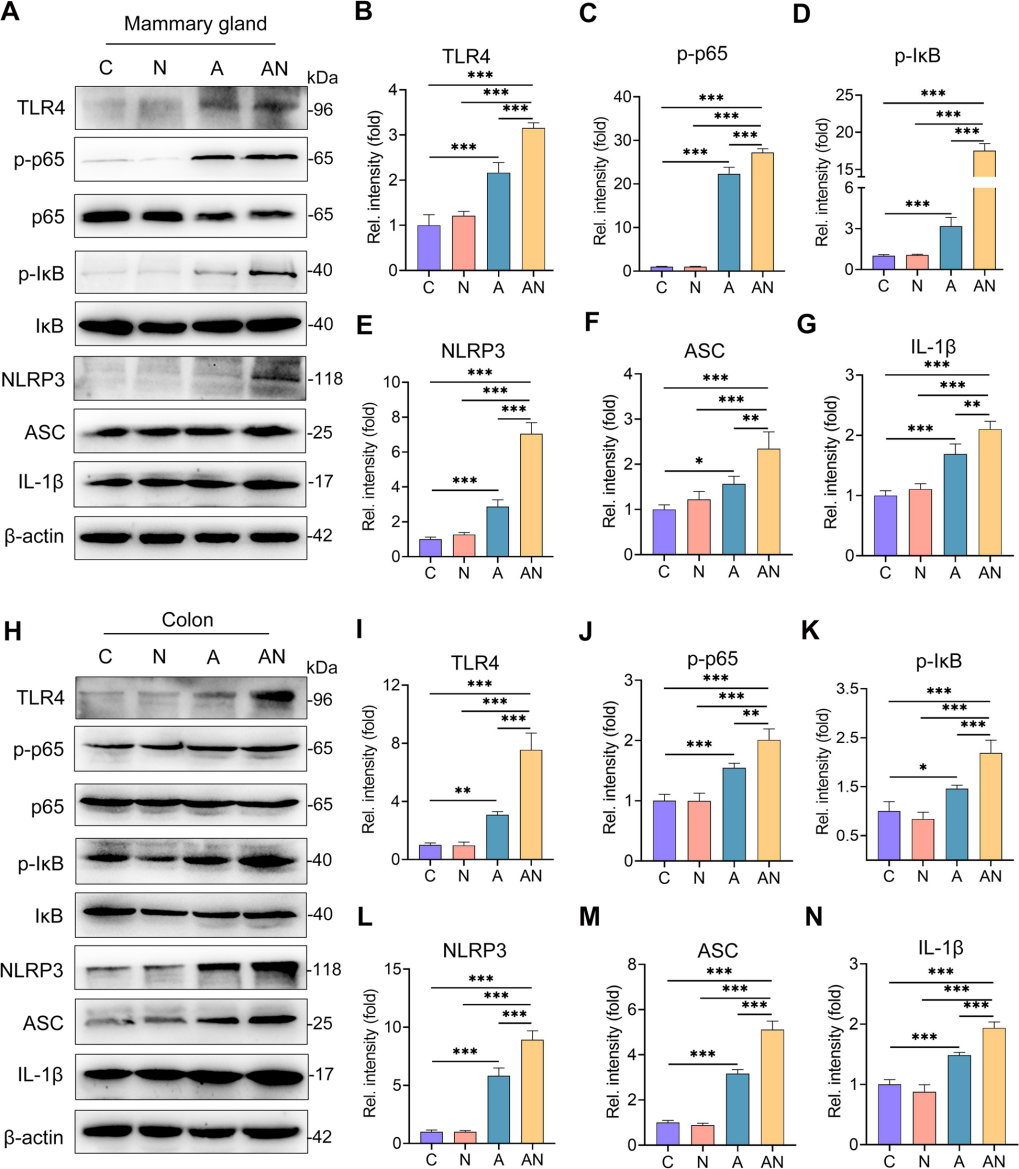
Sialic acid potentiates the activation of the TLR4-NF-κB/NLRP3 pathways caused by antibiotic in the mammary gland and colon. The protein levels of TLR4-NF-κB/NLRP3 pathways in mammary (**A**) and colonic (**H**) tissues from the indicated groups were determined by western blotting. The relative intensities of TLR4, p-p65, p-IκB, NLRP3, ASC, and IL-1β in the mammary gland (**B**–**G**) and colon (**I**–**N**) were determined (*n* = 4). Data are expressed as the mean ± SD (**B**–**G** and **I**–**N**) and one-way ANOVA was performed, followed by Tukey test (**B**–**G** and **I**–**N**). *p < 0.05, **p < 0.01, ***p < 0.001 indicate significance

Toll-like receptor (TLR)-4 is known to be responsible for the recognition of LPS [41], and thus mediates proinflammatory cytokine production by activating inflammatory signatures, such as NF-κB and NLRP3, which have been shown to participate in the pathogenesis of mastitis [22, 42]. Indeed, we found increased TLR4 levels in the A group compared with the C and N groups, which were enhanced in the AN group compared with the A group (Fig. 6A). Consistently, the A group had increased protein levels of p-p65, p-κB, NLRP3, ASC, and IL-1β in the mammary gland (Fig. 6A–G), indicating activation of the TLR4-NF-κB/NLRP3 axis. Similarly, this activation was potentiated in the AN group compared with the A group (Fig. 6A–G). In addition, we also detected increased activation of the TLR4-NF-κB/NLRP3 pathways in the colon tissue of the A group, which was consistently aggravated by SA supplementation (Fig. 6H–N). These results indicated that SA facilitates gut dysbiosis-triggered mastitis by potentiating the TLR4-NF-κB/NLRP3 signatures.

### SA facilitates antibiotic-induced gut dysbiosis

**Fig. 7:**
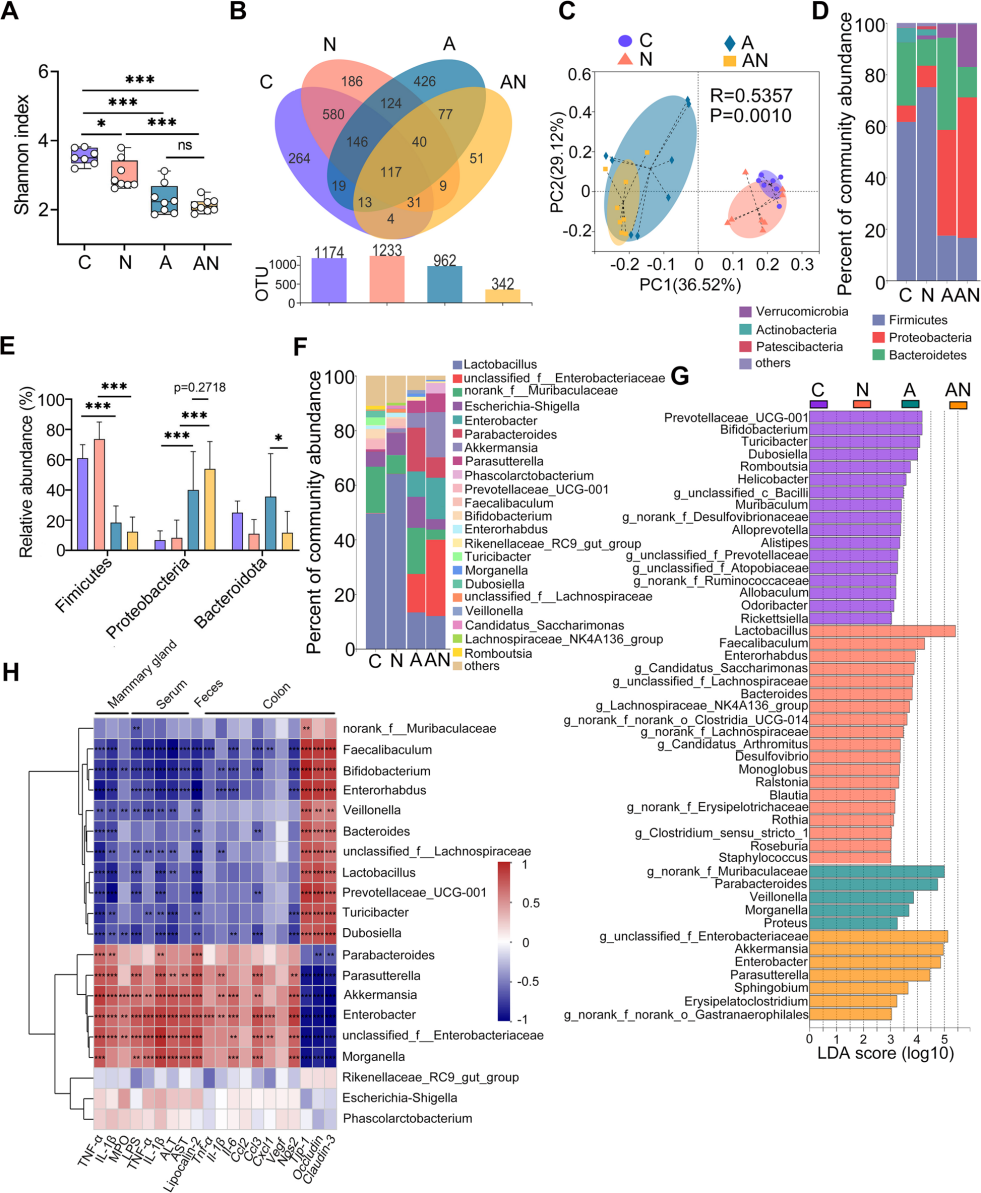
Sialic acid facilitates antibiotic-induced gut dysbiosis in mice. Mice were treated as described above and the fecal microbiota was assessed using 16S rDNA sequencing on day 14. **A** Shannon index showed that the N, A, and AN groups had a reduced alpha diversity (*n* = 7–8). **B** Venn analysis indicated the common and different OTUs in the different treatment groups (*n* = 7–8). **C** PCoA score plots for mouse fecal samples indicating the distinct intestinal microbiota structure (*R* = 0.5357, *P* = 0.001) from the different treated groups based on unweighted UniFrac distance (*n* = 7–8). **D** Bacterial composition at the phylum level from indicated groups (*n* = 7–8). **E** The relative abundances of *Firmicutes*, *Proteobacteria*, and *Bacteroidetes* in the different groups (*n* = 7–8). Data are expressed as the mean ± SD and one-way ANOVA was performed, followed by Tukey test. *p < 0.05, ***p < 0.001 indicate significance. ns, no significance. **F** Bacterial composition in genus level from indicated groups (*n* = 7–8). **G** LEfSe was performed to indicate the different bacterial taxa enriched in different treated groups (log_10_LDA score > 3). **H** Spearman correlation between gut microbiota and inflammatory parameters from the different groups. The red color denotes a positive correlation, while the blue color denotes a negative correlation. The intensity of the color is proportional to the strength of the Spearman correlation. *p < 0.05, **p < 0.01, ***p < 0.001 indicate significance. LEfSe, Linear discriminant analysis effect size

We further investigated the effects of SA on gut microbiota. Alpha-diversity indices, including Shannon, Chao 1 and ace, were markedly decreased in the N group, A group and AN group compared with the C group (Fig. 7A and Figure S9A, B). The AN group had lower alpha diversity indices than the A group, although no significant differences were detected (Fig. 7A and Figure S9A, B). The Venn analysis showed that the microbiota composition among the different groups was markedly different (Fig. 7B). The A group had fewer observed operational taxonomic units (OTUs) than the C group, and this decrease was enhanced in the AN group compared with the A group (Fig. 7B), indicating that SA promoted the decrease in gut microbial diversity caused by antibiotic. Principal coordinate analysis (PCoA) based on unweighted UniFrac distance and ANOSIM statistical analysis showed a separation in the gut microbiota structure among different groups (*R* = 0.5373, *p* = 0.001, Fig. 7C). At the phylum level, *Firmicutes*, *Proteobacteria* and *Bacteroidetes* were the predominant phyla in the fecal microbiota (false discovery rate (FDR) < 0.05) (Fig. 7D). The N group had increased *Firmicutes* and reduced *Bacteroidetes* compared with the C group (Fig. 7D, E). The A group had reduced *Firmicutes* but enriched *Proteobacteria* compared with the C group, while the AN group had higher *Proteobacteria* and *Verrucomicrobia* compared with the A group (Fig. 7D, E). At the family and genus levels, the microbiota compositions among different groups were markedly different (Fig. 7F and Figure S9C). Linear discriminant analysis effect size (LEfSe) was further performed to identify different bacterial taxa among the different groups (log_10_LDA score > 3). Moreover, we found that nineteen bacterial genera, including *Lactobacillus*, *Faecalibaculum*, *Enterorhabdus*, *Bacteroides*, *Ralstonia*, *Blautia*, and *Roseburia* were enriched in the N group (Fig. 7G). Seven bacterial genera were particularly abundant in the AN group, including *g__unclassified_f__Enterobacteriaceae*, *Akkermansia*, *Enterobacter*, *Parasutterella*, *Sphingobium*, and *Erysipelatoclostridium* (Fig. 7G), while the other five bacterial taxa were enriched in the A group (Fig. 7G). Additionally, Tax4Fun analysis revealed a significant difference of the metabolism pathways among the different groups (Figure S10). We observed several increases in potentially pathogenic bacterial pathways and aerobic respiration-associated pathways in the A group, including *Staphylococcus aureus* infection, nitrogen metabolism and oxidative phosphorylation [43], and these increases were enhanced in the AN group (Figure S10). Furthermore, Spearman correlation analysis was performed to identify the association between different key genera and mucosal, systemic and mammary inflammatory parameters, or colon TJ proteins profiles. The results showed that microbes enriched in the AN group, including *g__unclassified_f__Enterobacteriaceae*, *Akkermansia*, *Enterobacter* and *Parasutterella*, were significantly correlated with most proinflammatory parameters and TJs profiles (Fig. 7H). The correlation coefficients for other genera enriched in C and N groups, such as *Prevotellaceae*_UCG-001, *Bifidobacterium*, *Lactobacillus*, *Bacteroides*, and *Faecalibacterium* were the opposite (Fig. 7H). These results demonstrated marked changes in the composition and function of the intestinal microbiota after SA supplementation, indicating the essential role of gut microbes in the SA-exacerbated inflammatory responses.

### FMT from the A or AN group mimics mastitis in recipient mice

**Fig. 8:**
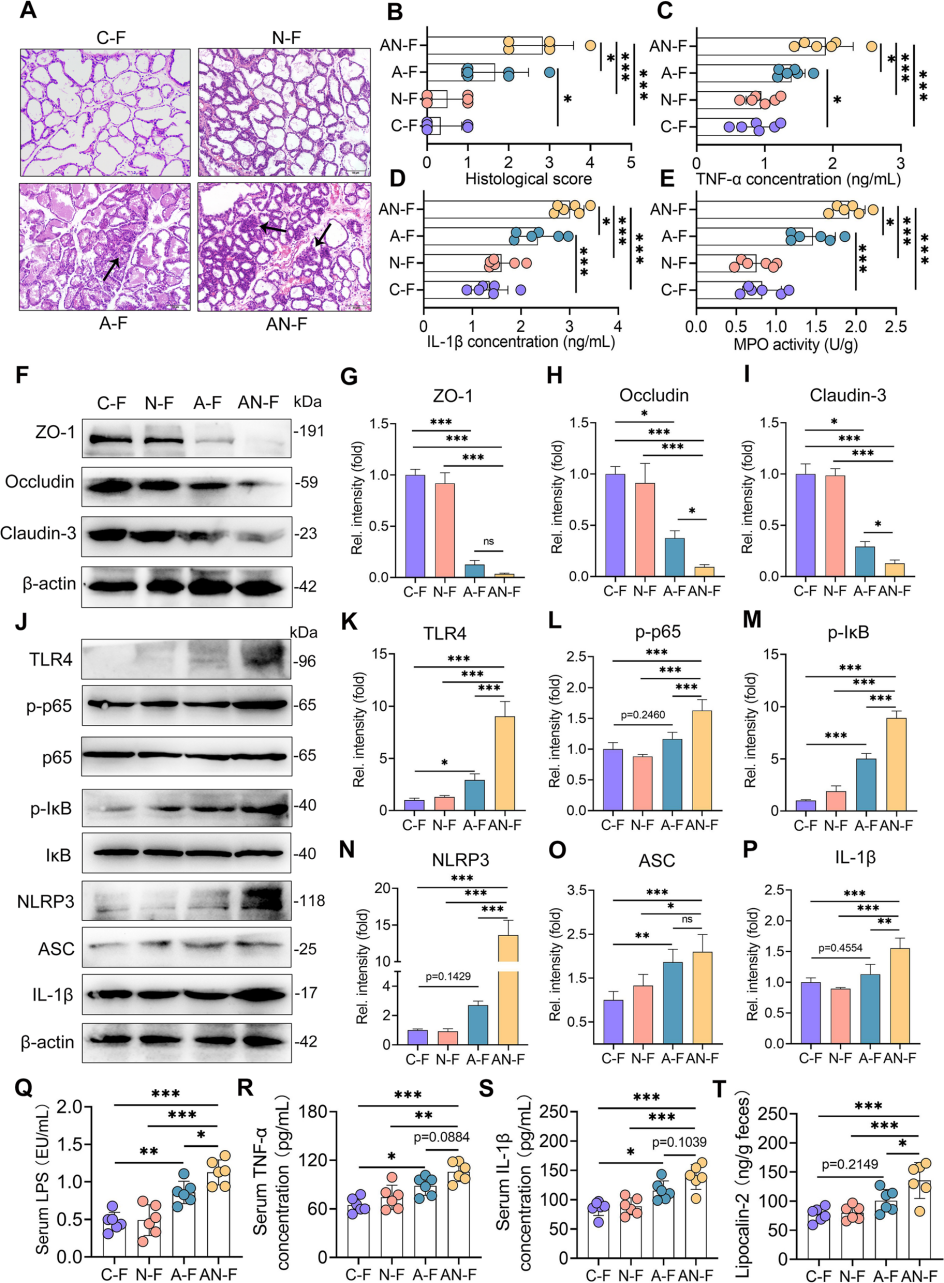
FMT from AN group mice induces mastitis in recipient mice.** A** Representative images of H&E-stained sections from different FMT groups (scale bar, 50 μm). Arrows indicate inflammatory infiltration.** B** The histological scores of mammary glands from the different FMT groups (*n* = 6). The proinflammatory markers TNF-α (**C**) and IL-1β (**D**) and MPO activity (**E**) were measured (*n* = 6). **F** Representative images of ZO-1, Occludin, and Claudin-3 in mammary glands from the indicated mice. The intensities of ZO-1, Occludin, and Claudin-3 were determined (**G**–**I**) (*n* = 4). **J** The protein levels of the TLR4-NF-κB/NLRP3 pathways in the mammary gland were assessed by western blotting. The relative intensities of TLR4, p-p65, p-IκB, NLRP3, ASC, and IL-1β in the mammary gland were determined (**K**–**P**) (*n* = 4). Serum LPS (**Q**), TNF-α (**R**), IL-1β (**S**), and fecal lipocalin-2 (**T**) were determined (*n* = 6). Data are expressed as the mean ± SD (**B**–**E**,** G**–**I**,** K**–**P,** and **Q**–**T**) and one-way ANOVA was performed, followed by Tukey test (**B**–**E**,** G**–**I**,** K**–**P,** and **Q**–**T**). *p < 0.05, **p < 0.01, ***p < 0.001 indicate significant difference

Next, through FMT, we investigated whether SA facilitates gut dysbiosis-induced mastitis by regulating the gut microbiota. Interestingly, FMT from donors of the A group (A–F) induced mammary injury and increased inflammatory marker levels in recipient mice compared with FMT from the C or N group (C-F or N-F) (Fig. 8A–E), while FMT from the AN group FMT (AN-F) resulted in higher mastitis parameters in the mice compared with the A-F group (Fig. 8A–E). Furthermore, we showed that A-F damaged the blood-milk barrier integrity by reducing the TJ proteins ZO-1, Occludin, and Claudin-3 compared with the C-F or N-F groups (Fig. 8F–I). Similarly, these decreases were potentiated in AN-F mice compared with A-F mice (Fig. 8F–I). In addition, the A-F mice had increased activation of TLR4-NF-κB/NLRP3 signatures in the mammary gland compared with the C-F or N-F group, which was enhanced in AN-F mice compared with the A-F mice (Fig. 8J–P). Consistently, we also showed increased mucosal and systemic inflammation in the A-F group compared with the C-F or N-F group, and these increases were aggravated in the AN-F group compared with the A-F group (Fig. 8Q–T). We then showed that the N-F and AN-F group had increased SA levels compared with the C-F or A-F group, respectively. A-F group also showed higher SA levels than C-F group (Figure S7B). These data indicated that FMT from mice in the AN group induced mastitis in recipient mice, which may depend on distinct microbial compositions and functions.

### SA boosts pathogenic bacterial growth and virulence gene expression, aggravating pathogen-induced proinflammatory cytokine production and mastitis

**Fig. 9:**
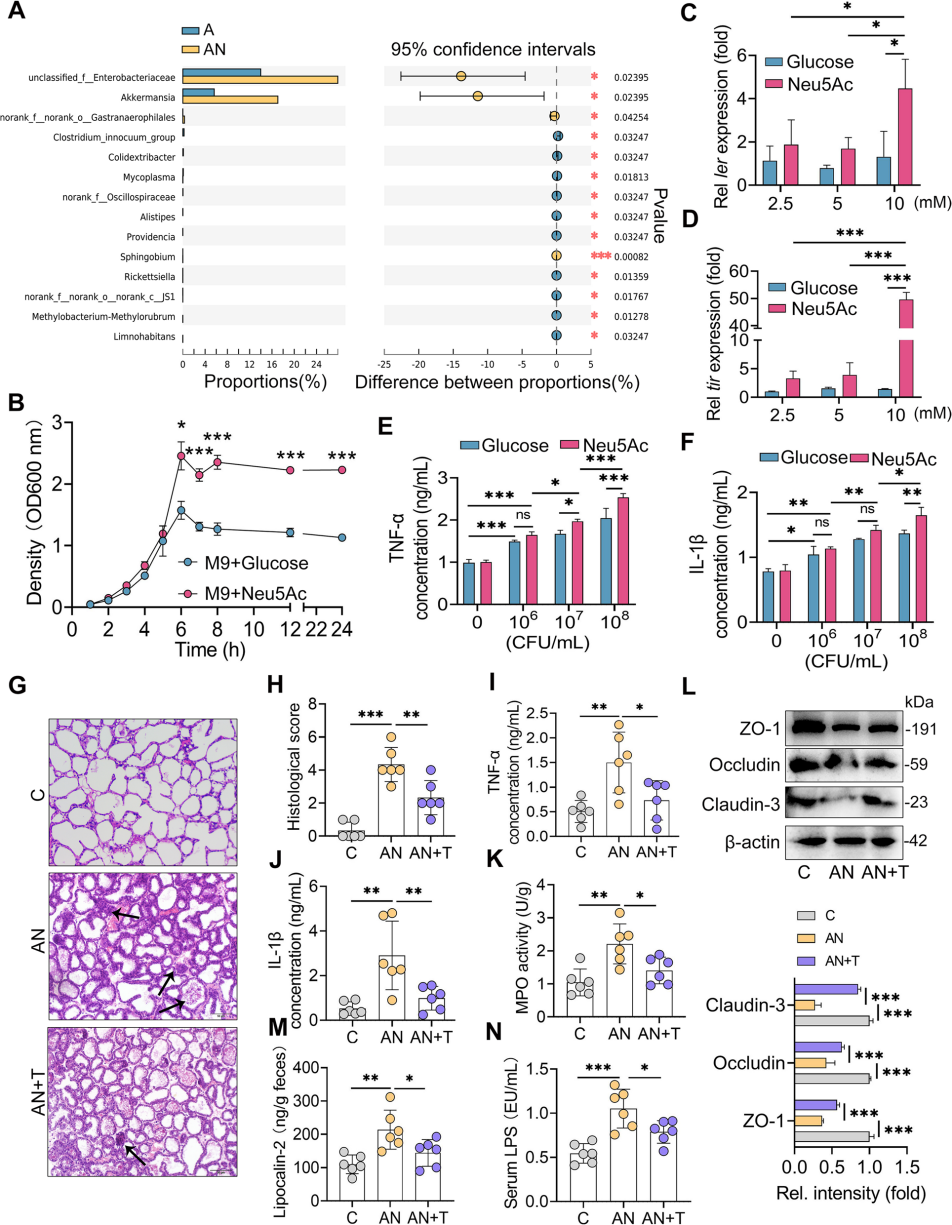
Sialic acid facilitates mastitis by promoting the growth and virulence of *Enterobacteriaceae*.** A** A Wilcoxon rank-sum test was performed to identify the differential bacterial taxa in the A and AN groups (FDR < 0.05) (*n* = 8). **B** The effect of different monosaccharides on the growth of *E. coli* was determined in M9 medium (*n* = 3). **C**,** D**. *E. coli* was cultured with 2.5, 5, or 10 mM Neu5Ac or glucose for 6 h, and the relative mRNA levels of *ler* (**C**) and *tir* (**D**) were measured using qPCR (*n* = 3). **E**,** F**. *E. coli* was cultured with 10 mM Neu5Ac or glucose for 6 h, and then macrophages (10^6^ cells/mL in 6-well plates) were treated with different concentrations of *E. coli* for 24 h. The proinflammatory cytokines TNF-α (**E**) and IL-1β (**F**) were measured by ELISA (*n* = 3). **G** Representative images of H&E-stained mammary glands from the indicated groups (scale bar, 50 μm). **H** Histological scores of the mammary gland. TNF-α (**I**), IL-1β (**J**), MPO (**K**), fecal lipocalin-2 (**M**), and serum LPS (**N**) levels were determined (*n* = 6). **L** The TJ proteins ZO-1, Occludin, and Claudin-3 were assessed by western blotting (n = 4). Data are expressed as the mean ± SD (**C**–**F**,** H**–**K**, and **M**–**N**). Statistical significance in the same time was determined by Student’s *t* test (**B**). For **C**–**F**, two-way ANOVA was performed for statistical analysis (**C**–**F**). For **H**–**K** and **M**–**N**, one-way ANOVA was performed, followed by Tukey test. *p < 0.05, **p < 0.01, ***p < 0.001 indicate significance. ns, no significance. FDR, false discovery rate; T, sodium tungstate

Considering that the AN group had higher proinflammatory markers and gut microbiota disruption than the A group and that FMT mimicked the proinflammatory effects of SA, we then identified the potential microbes derived from SA by performing a Wilcoxon rank-sum test bar plot on the genus levels (FDR < 0.05) of the A and AN groups. Consistently, the AN group had increased *g__unclassified_f__Enterobacteriaceae* and *Akkermansia* (Fig. 9A). Previous studies have reported that *Akkermansia* can use mucosal polysaccharides as nutrients [44]. We further investigated whether SA can be used as a carbon source for *Enterobacteriaceae* bacteria by assessing the growth of *E. coli*, a typical *Enterobacteriaceae* species, in vitro by supplementing M9 medium with 10 mM Neu5Ac or glucose. The results revealed that Neu5Ac-treated *E. coli* LF82 cells had a higher cell density than those cells treated with glucose, indicating that SA was a preferential source of energy for *E. coli* (Fig. 9B). Moreover, increased virulence gene levels in *E. coli* LF82, including *ler* and *tir* (locus of enterocyte effacement 1 and 5) [45], were detected in SA-treated *E. coli* LF82 compared to glucose-treated cells (Fig. 9C, D). In addition, SA-treated *E. coli* LF82 induced higher proinflammatory cytokine levels on macrophages than glucose-treated *E. coli* LF82 in vitro (Fig. 9E, F).

To confirm the role of *Enterobacteriaceae* in SA-mediated mastitis, sodium tungstate, a specific inhibitor of the expansion of *Enterobacteriaceae* during episodes of inflammation [46, 47], was performed in AN-treated mice. We first confirmed that sodium tungstate treatment (AN + T) reduced intestinal *Enterobacteriaceae* but had little influence on other genera (Figure S11A-B). Tungstate treatment ameliorated mastitis compared with that in the AN group, as evidenced by improved mammary injury and reduced levels of proinflammatory parameters (Fig. 9G–K). In addition, the AN + T mice had a restored blood-milk barrier integrity with increased TJ proteins compared with that of the AN group (Fig. 9L). Moreover, sodium tungstate treatment also alleviated AN-triggered mucosal and systemic inflammation in mice (Fig. 9M, N). Altogether, these results indicated that SA serves as a preferential carbon source for pathogens to promote pathogen expansion and virulence gene expression and subsequently trigger a more severe inflammatory response.

### Commensal L. reuteri alleviates SA-aggravated mastitis in mice

**Fig. 10:**
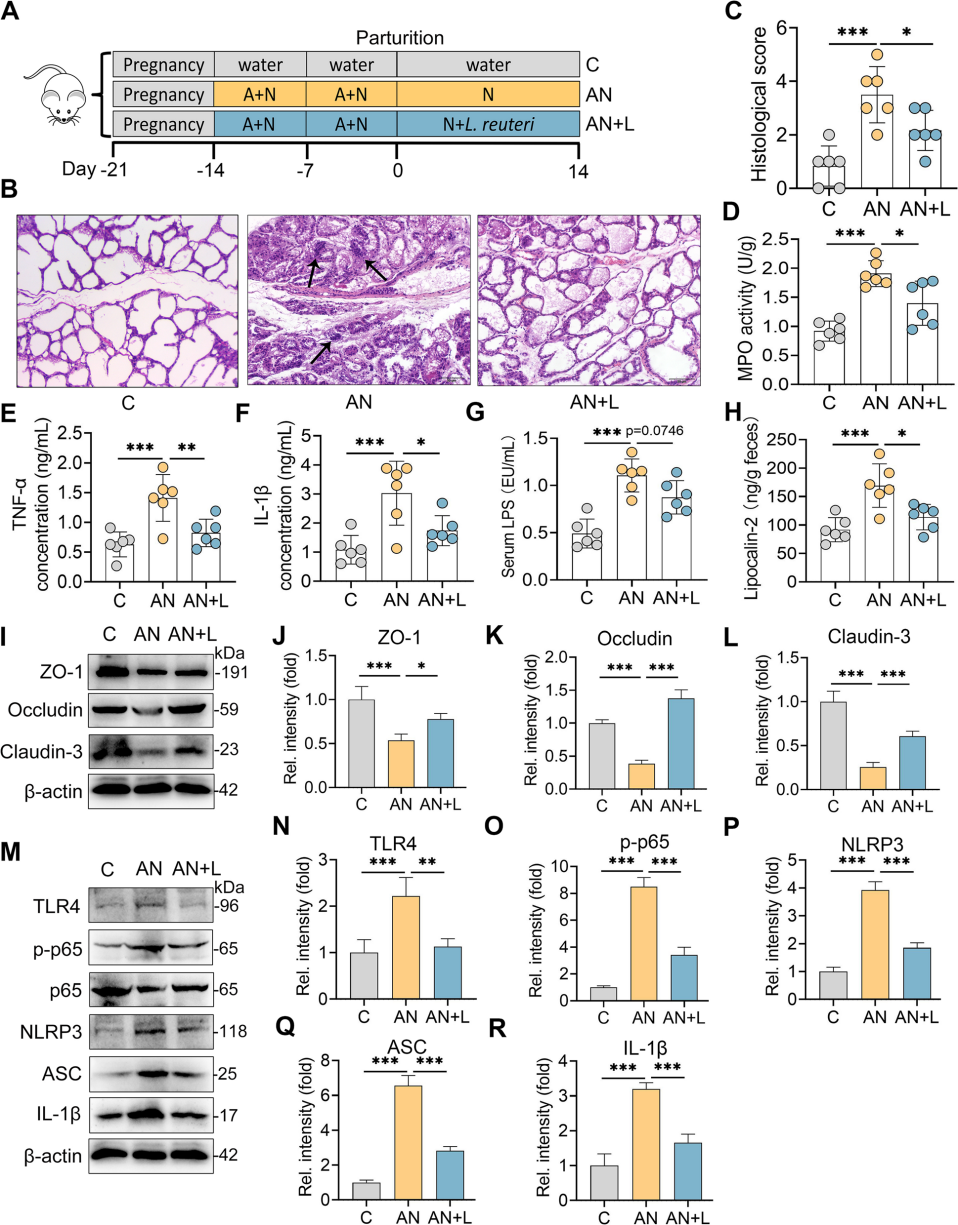
*L. reuteri* ameliorates sialic acid-facilitated mastitis in antibiotic-treated mice. **A** Schematic representation of *L. reuteri* supplementation. Mice were treated with ampicillin and Neu5Ac for 14 days before parturition, followed by the removal of ampicillin and treatment with *L. reuteri* (10^9^ CFU/mouse per day) for the next 14 days. **B** Representative H&E-stained sections of mammary glands from different groups (scale bar, 50 μm). **C** Histological scores of the mammary sections (*n* = 6). **D**–**H**. Mammary MPO (**D**), TNF-α (**E**), IL-1β (**F**), serum LPS (**G**), and fecal lipocalin-2 (**H**) levels were assessed (*n* = 6).** I**. Representative images of ZO-1, Occludin, and Claudin-3. **J**–**L**. The relative intensities of mammary ZO-1 (**J**), Occludin (**K**), and Claudin-3 (**L**) were determined (*n* = 4). **M** The protein levels of TLR4-NF-κB/NLRP3 pathways were determined by western blotting. **N**–**R** Relative intensities of mammary TLR4 (**N**), p-p65 (**O**), NLRP3 (**P**), ASC (**Q**), and IL-1β (**R**) were determined (*n* = 4). Data are expressed as the mean ± SD (**C**–**H**,** J**–**L**, and **N**–**R**) and one-way ANOVA was performed, followed by Tukey test (**C**–**H**,** J**–**L**, and **N**–**R**). *p < 0.05, **p < 0.01, ***p < 0.001 indicate significance

Considering the increase in *Lactobacillus* in the N group and its depletion in the A and AN groups (Fig. 7F–G), as well as its correlation with mastitis parameters (Fig. 7H), we therefore investigated the protective role of commensal *Lactobacillus* on AN-mediated mastitis by treating mice with *L. reuteri* (Fig. 10A), a probiotic that has been reported to alleviate *E. coli*-induced mastitis in mice [22]. We found that *L. reuteri* treatment (AN + L) improved mammary leucocyte infiltration, proinflammatory marker levels and barrier integrity compared those of the AN group (Fig. 10B–F). Moreover, we showed that the AN + L group had reduced serum LPS and fecal lipocalin-2 levels compared with the AN group (Fig. 10G, H), indicating improved systemic and mucosal inflammation. In addition, we found that *L. reuteri* restored the mammary ZO-1, Occludin and Claudin-3 levels reduced by SA treatment (Fig. 10I–L). Furthermore, we showed that *L. reuteri* treatment reduced the activation of TLR4-NF-κB/NLRP3 signatures compared with those of the AN group (Fig. 10M–R). Collectively, these results indicate that commensal *L. reuteri* alleviates AN-induced mastitis in mice.

### SARA cows have different ruminal microbial compositions and sialidase inhibitor alleviates mastitis in mice caused by RMT for SARA cows (SRMT)

**Fig. 11:**
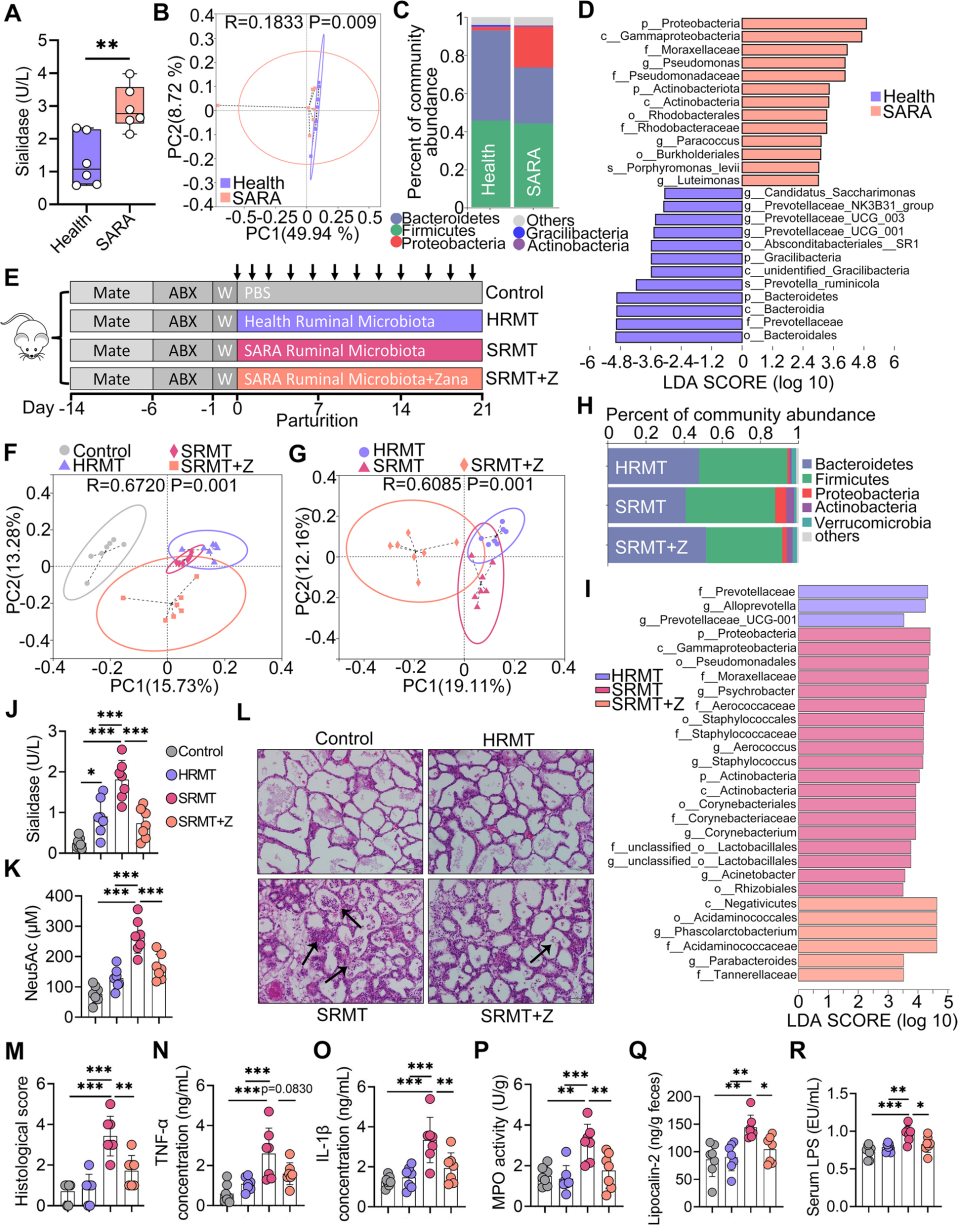
Sialidase inhibitor alleviates SRMT-induced mastitis and systemic inflammatory responses in mice. **A** The ruminal sialidase levels from indicated groups (*n* = 6). **B**. PCoA showed distinct ruminal microbial structure (*R* = 0.1833, *P* = 0.009) between health and SARA groups based on unweighted UniFrac distance (*n* = 6). **C** Microbial compositions at the phylum level from indicated groups (*n* = 6). **D** LEfSe analysis identified the different taxa in different treated groups (log_10_LDA score > 3) (*n* = 6). **E** RMT experimental protocol. Mice were treated with antibiotics (200 mg/kg ampicillin, metronidazole and neomycin and 100 mg/kg vancomycin) daily through oral gavage for 5 days. Furthermore, mice were orally gavaged with 300 μL of ruminal supernatant for three consecutive days after antibiotics removal and once every 2 days thereafter for 19 days. The control mice were orally gavaged with an equal volume of sterile PBS. SRMT + Z group was treated with zanamivir (0.5 mg/mL in drinking water) simultaneously for 21 days (*n* = 7). **F** PCoA showed successful ruminal microbial colonization after RMT based on unweighted UniFrac distance (*R* = 0.6720, *P* = 0.001, *n* = 7). **G** PCoA showed distinct ruminal microbial structure between different RMT groups based on unweighted UniFrac distance (*R* = 0.6085, *P* = 0.001, *n* = 7). **H** Microbial compositions at the phylum level in different treated groups (*n* = 7). **I**. LEfSe analysis identified the different taxa in different RMT groups (log_10_LDA score > 3.5) (*n* = 7). **J**,** K** The intestinal sialidase and Neu5Ac levels from different treated groups (*n* = 7).** L** Representative H&E-stained sections of mammary glands from the indicated mice (scale bar, 50 μm). **M** Histological scores of mammary tissues from the different treatment groups (*n* = 7). Inflammatory markers of mammary TNF-α (**N**), IL-1β (**O**) and MPO activity (**P**), and fecal lipocalin-2 (**Q**) and serum LPS (**R**), and were assessed by ELISA (*n* = 7). Data are expressed as boxplot (**A**) or the mean ± SD (**J**–**K** and **M**–**R**). A Student’s *t* test (**A**) or one-way ANOVA was performed, followed by Tukey test (**J**–**K** and **M**–**R**). *p < 0.05, **p < 0.01, ***p < 0.001 indicate significance. Z, zanamivir

Microbial sialidase plays an essential role in the release of SA from the host. We next showed that SARA cows had increased microbial sialidase levels in the rumen compared with those in the health group (Fig. 11A). Consistently, SARA cows had ruminal microbial structure distinct from the health group using the unweighted UniFrac distance (*R* = 0.1833, *P* = 0.009, Fig. 11B). Alpha diversity analysis showed that no significant differences were detected in the richness and diversity of the ruminal microbiota between SARA and health group (Figure S12A–D). At the phylum level, SARA cows had enriched *Proteobacteria* and depleted *Bacteroidetes* compared with those of health group (Fig. 11C). At the family level, *Moraxellaceae* was enriched and *Prevotellaceae* was depleted in the SARA group compared to the health group (Figure S12E), and these differences were confirmed by LEfSe (log_10_LDA score > 3, Fig. 11D). We then investigated whether ruminal dysbiosis contributed to the development of mastitis and the extent of SA in SARA-associated mastitis by using SRMT accompanied by treatment with zanamivir, a specific sialidase inhibitor [33]. The RMT protocol was performed and modified according to previous studies [46, 48, 49] (Fig. 11E). We first confirmed that the gut microbial structure of control group was significantly separated from the RMT groups by PCoA (*R* = 0.6720, *P* = 0.001), indicating successful ruminal microbial colonization (Fig. 11F). Venn diagram analysis also supported this finding by showing higher OTUs in RMT groups than the control group (Figure S12F). Moreover, RMT from different donors and with or without sialidase inhibitor treatment induced different gut microbial structure (*R* = 0.6085, *P* = 0.001, Fig. 11G). Consistent with the donors, no significant differences were determined among the different recipient mice (Figure S12G–I). At the phylum level, similar to the SARA cows, SRMT group had enriched *Proteobacteria* and depleted *Bacteroidetes* compared with those of the RMT from healthy cows (HRMT) group, while these increases were altered after zanamivir treatment (Fig. 11H). At the family level, *Moraxellaceae* was enriched and *Prevotellaceae* was depleted in the SRMT group compared to the HRMT group but reduced by zanamivir treatment (Figure S12J). LEfSe also confirmed *Proteobacteria* and *Moraxellaceae* were enriched in the SRMT group (log_10_LDA score > 3.5, Fig. 11I), which have been reported as the SA-utilizing opportunistic pathogen [50]. Indeed, SRMT increased intestinal sialidase levels compared with those of the control or HRMT groups, while zanamivir treatment reduced sialidase expression compared with that of the SRMT group (Fig. 11J), with consistent intestinal SA levels (Fig. 11K). The histological analysis showed that the SRMT group, but not the HRMT group, had marked inflammatory changes as leukocyte infiltration and hyperplasia, and these changes were ameliorated by zanamivir treatment (Fig. 11L, M). Moreover, we found that the SRMT group had several increases in mastitis parameters, including MPO activity, TNF-α, and IL-1β levels, compared with the control and HRMT groups (Fig. 11N–P); however, zanamivir treatment alleviated these increases compared with those of the SRMT group (Fig. 11N–P). Furthermore, we showed that the SRMT group had increased linpocalin-2 levels compared with the control and HRMT groups, and zanamivir treatment alleviated SRMT-induced mucosal inflammation (Fig. 11Q).Additionally, increased systemic inflammatory markers, including serum LPS, TNF-α, and IL-1β levels, were detected in the SRMT groups compared with control and HRMT groups (Fig. 11R and S12K-L), while these markers were reduced in the SRMT + Z group compared with the SRMT group (Fig. 11R and Figure S12K, L), indicating that the sialidase inhibitor alleviated SRMT-induced systemic immune imbalance. To confirm the role of gut dysbiosis in the pathogenesis of mastitis, and the regulatory effect of diet, we supplemented RMT mice with a high-starch diet (HSD) to simulate a HGD in cows [48]. SRMT group with a HSD treatment had higher intestinal SA levels compared with the other groups or SRMT without a HSD treatment (Fig. 11K and Figure S13A). Consistently, the SRMT group with a HSD treatment, but not the control or HRMT group, showed induction of significant mastitis, while zanamivir treatment alleviated SRMT-associated mastitis (Figure S13B–F). Collectively, our results show that sialidase inhibitor ameliorate SRMT-induced mastitis, systemic inflammation, and opportunistic pathogen expansion.

## Discussions

Milk is essential for people’s normal lives because it provides abundant nutrients and trace elements, which has led to milk yield and quality serving as markers of national agricultural modernization [51]. Mastitis is the most severe disease that decreases milk yield and quality, which results in huge economic losses every year throughout the world [52]. Although pathogen invasion of the mammary gland, especially by bacteria including Gram-positive *Streptococcus* and *S. aureus* and Gram-negative *E. coli* and *Klebsiella*, has been thought to be the major cause of mastitis [53, 54], an increasing number of studies have suggested that gut microbiota play a crucial role in the pathogenesis of mastitis. Our previous results suggested that gut dysbiosis caused by antibiotics can initiate mastitis and promote its development upon pathogen invasion in mice [21, 22]. Moreover, cows with mastitis showed distinct gut microbiota profiles, and FMT from cow to mouse induced mastitis in mice [19, 20]. In dairy cows, SARA induced by the excessive consumption of a HGD increased the risk for mastitis [27, 55], but little is known about how SARA-mediated metabolic changes contribute to mastitis pathogenesis and its influence on microbiota in turn. In the present study, we revealed that SARA induced marked metabolic changes in the rumen. Among the most enriched metabolites in SARA cows was SA, which was correlated with mastitis parameters. Treatment with SA exacerbates mastitis in antibiotic-treated mice through the microbiota-gut-mammary axis by facilitating the growth of opportunistic pathogen and activating TLR4-NF-κB/NLRP3 signatures, while limitation of SA production, regulation of gut homeostasis by commensal microbes and targeted inhibition of opportunistic pathogen expansion attenuated gut dysbiosis-associated mastitis in mice.

Evidence supporting a causal role for host or microbiota-associated metabolites in the pathogenesis of distal diseases has been reported. For example, microbial 4-EPS causes anxiety behavior by impairing oligodendrocyte maturation and reducing oligodendrocyte-neuron interactions [14]. Trimethylamine derived from the gut microbiota was found to be elevated in alcohol-associated hepatitis and contributes to ethanol-induced liver injury in mice [56]. Moreover, gut microbiota produced delta-valerobetaine facilitates Western diet-induced obesity by depressing mitochondrial fatty acid oxidation [57]. Gao et al. showed that SARA cows demonstrate alternations in plasma metabolites, including β-hydroxybutyrate, non-esterified fatty acids, cholesterol and triglyceride, which are associated with a change in oxidative stress levels [58]. Another study found that a grain-based SARA increases serum LPS levels and triggers systemic inflammation [59]. Although SARA has been reported to shape the systemic metabolic profile and is associated with the development of mastitis, whether a ruminal dysbiosis-derived metabolite, such as SA, can lead to mastitis has not been investigated.

SAs exist widely on the surface of vertebrate cells, especially on mucosal surfaces [32], where SAs directly regulate the interaction between microbes and the host. Hence, the elevated concentration of SAs in SARA cows potentially does not originate directly from foods and agrees with previous findings that there is a low free SA level in the gut under physiological conditions [34]; however, increased SA levels were observed in intestinal inflammation and gut dysbiosis by increasing levels of sialidase [33, 34]. Indeed, increased gastrointestinal inflammation by enriched LPS levels and distinct microbiota compositions have been reported in SARA cows [27, 55]. In the current study, we confirmed that the increased SAs contributed to gut dysbiosis-associated mastitis, as evidenced by SA supplementation in conventional mice showing no obvious effect on mastitis in mice. In antibiotic-treated mice, however, SA consumption aggravated antibiotic-induced mammary inflammatory responses, indicating that SA facilitated the development of mastitis and that this effect depended on the gut microbiota. Previous studies have shown that SA can trigger an inflammatory response when it encounters circulating anti-SA antibodies [60]. Moreover, increased serum Neu5Ac levels were associated with myocardial injury [61]. The gap between our findings and previous studies may be attributed to distinct experimental conditions or animal models. In addition, SA-antibiotic-treated mice had increased mucosal and systemic inflammation, accompanied by damaged gut barrier functions and increased serum LPS levels, which has been associated with remote diseases through TLR4 [41]. Increased TLR4 levels were detected in the colon and mammary gland of AN mice, accompanied by enhanced downstream NF-κB and NLRP3 pathways, which are involved in mastitis pathogenesis and other gut dysbiosis-associated diseases [22, 42, 62, 63]. These results suggest that SA facilitates the production of gut dysbiosis-derived LPS, enabling the development of mastitis through the microbiota-gut-mammary axis by TLR4-mediated signatures [63].

FMT has been widely used to verify the causative relationship between gut microbiota changes and disease outcomes [19, 21, 64]. We showed that FMT from the A or AN group induced mastitis in recipient mice, suggesting that gut dysbiosis is attributed to the development of mastitis [19, 21, 22]. Our results agree with previous findings that microbiota-liberated host SA facilitates enteric pathogen invasion and promotes host gene mutant-induced opportunistic pathogen expansion [34, 65, 66]. Similar findings have also been reported in other microbiota-liberated host sugars, including succinate and fucose [45, 67], indicating that changed carbon metabolism may confer an expansion advantage for pathogens. It has been reported that intestinal inflammation can induce gut dysbiosis and promote the overgrowth of *Enterobacteriaceae* [68], which was also observed in dysbiosis-induced mastitis mice treated with antibiotics [21]. Our findings can be extended to a previous study of the inhibition of SA release by the knockout of the α2,3 sialyltransferase *St3gal4*, which reduced *Enterobacteriaceae* abundance and alleviated colitis in DSS-treated mice [69]. In vitro experiments showed that SA served as a preferential energy source for pathogens, which further promoted bacterial growth, enhanced the virulence and triggered inflammation. *E. coli* contains a SA transporter (*nanT*) and aldolase/lyase (*nanA*), which transports free SA and subsequently catabolizes SA into pyruvate and N-acetylmannosamine respectively [70]. Similar results have been reported that host-derived sugars promote pathogen growth and virulence [45, 71]. Inhibition of *Enterobacteriaceae* by sodium tungstate improved mastitis, which indicates that target depletion of opportunistic pathogen during gut dysbiosis is a potential strategy to alleviate gut and parenteral disease [47, 72]. Interestingly, although the N group also had a changed microbiota composition including enriched *Lactobacillus*, *Faecalibaculum*, *Enterorhabdus*, *Bacteroides*, *Blautia* and *Roseburia*, no inflammatory changes were detected, which implies the beneficial role of commensal microbiota on the metabolism of SA [34]. Among these, *Lactobacillus* has been identified as an effective SA-utilizing bacterium [73]. Other microbes enriched in SA-treated conventional mice including *Faecalibaculum*, *Bacteroides*, *Blautia* and *Roseburia*, are well known as SCFA producers [74–77], which suggest that SA may act as a nutrient to promote commensal microbes to produce SCFA, but further verification is needed. Interestingly, mice supplemented with *L. reuteri*, a probiotic that can improve *E. coli*-induced mastitis [22], alleviated SA-facilitated mastitis in antibiotic-treated mice. However, whether the beneficial role of *L. reuteri* is attributed to the effective capacity of SA metabolism or its immune regulation ability remains unknown. Together, these results suggest that different gut microbial contents may confer distinct results on host SA metabolism and highlight the importance of maintaining gut homeostasis for the prevention of mastitis.

SAs are rarely synthesized by microbes and are commonly derived from host glycans by sialidase to become freely available for commensal or pathogenic bacteria [70, 78]. In this study, we showed that SARA cows had increased ruminal microbial sialidase, accompanied by a distinct ruminal microbiota structure. A previous study showed that SARA cows had increased *Ruminococcus* levels [48], which was reported to release SA from sialyllactose and promote bacterial adaptation to the intestine [79]. Commensal *Bacteroides* encode sialidase but lack the catabolic pathway [34, 70]. Thus, this genus commonly facilitated other pathogens expansion in the gut, including *Salmonella typhimurium*, *Clostridioides difficile* and *E. coli* [34, 69]. In the present study, ruminal SA-utilizing commensal *Prevotellaceae* is depleted [73], while the SA-utilizing opportunistic pathogenic *Moraxellaceae* is enriched in SARA cows [50], which supports that abnormal SA metabolism facilitates microbial dysbiosis. Notably, *Moraxellaceae* also enriched in the rumen of clinical and subclinical mastitis cows [49, 80], implying its pathogenic effect in mastitis pathogenesis but further research is needed. Consistent with our results that zanamivir treatment reduced *Moraxellaceae* abundance and SRMT-mediated mastitis, Ahmed et al. found that inhibition of sialidase can limit *Moraxellaceae*-induced inflammation [81]. However, the origin of microbial sialidase and how it is elevated are unclear in the present study. We confirmed that inhibition of SA release alleviated SARA-induced mastitis by zanamivir, which could be extended to previous studies showing that inhibition of sialidase is a potential strategy to improve gut dysbiosis and subsequent inflammatory responses [66, 69]. Another potential reason for the increased SA is mucosal inflammation, in which LPS triggered host neuraminidase release through TLR4 and treatment with sialidase inhibitor attenuated inflammation caused by LPS or pathogen invasion [33, 82]. Although the specific mechanism for the pathogenesis of SARA-induced mastitis has not been proven, ruminal microbiota disruption and inflammation may act synergistically. Hence, the protective effects of sialidase inhibition may work in a variety of ways, and sialidase-deficient mice will be used in the future to determine the role of host sialidase in mastitis pathogenesis and gut microbiota. Our findings did not allow us to rule out the pathological roles of other metabolites. Likewise, it is still unclear which certain species of *Enterobacteriaceae* are the key taxa responsible for mastitis pathogenesis. Moreover, further studies are needed to gain greater insight into other potentially harmful metabolites on the pathogenesis of mastitis and the protective effects of metabolites depleted in SARA cows.

**Fig. 12:**
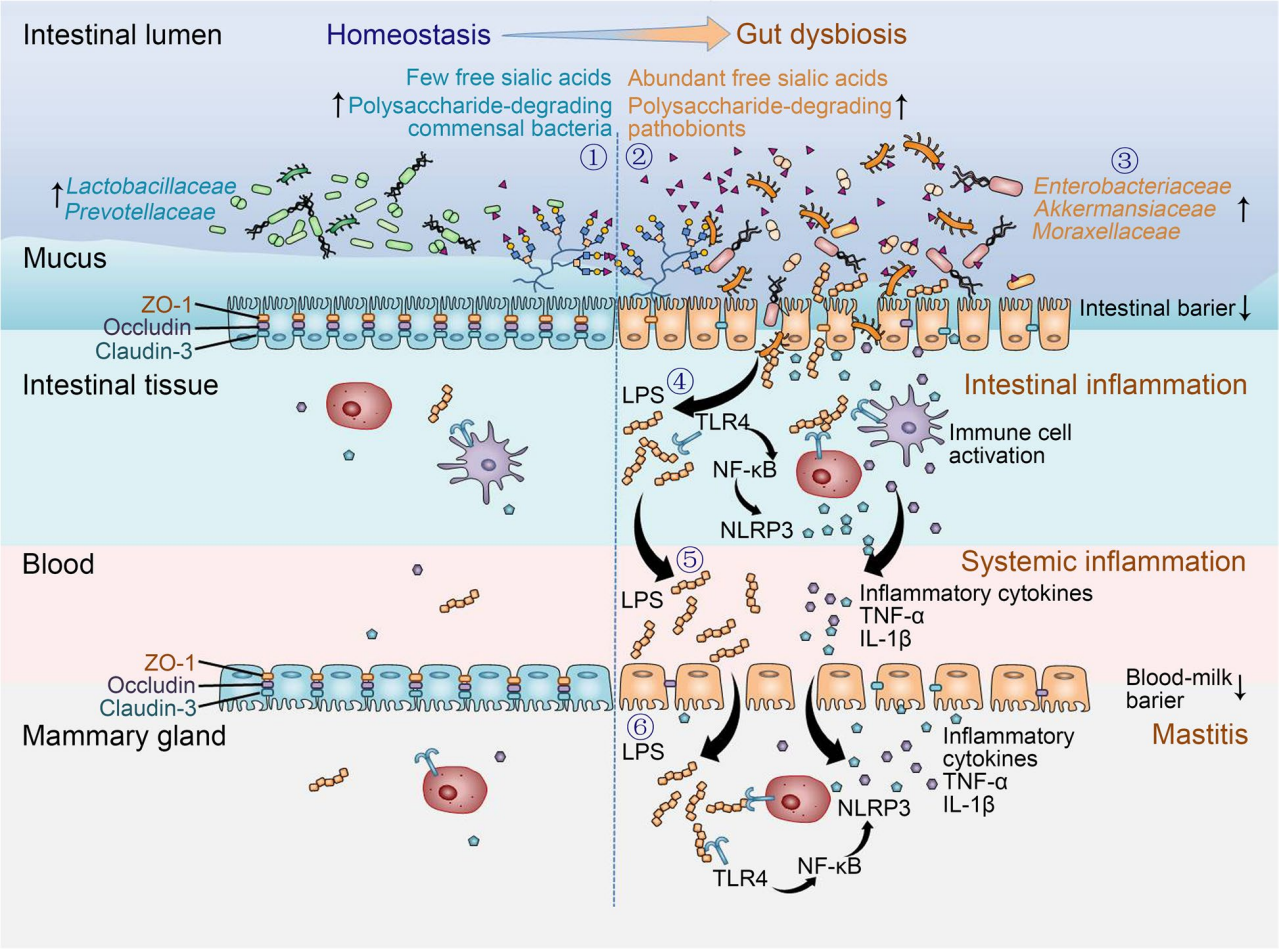
Schematic illustration of the mechanism by which SA facilitates gut dysbiosis-induced mastitis through the microbiota-gut-mammary axis. **①** Under gut homeostasis conditions, sialic acids bind to mucus, with few free SAs present in the intestinal lumen, which are then degraded by polysaccharide-degrading commensal bacteria (e.g., *Lactobacillaceae* and *Prevotellaceae*). **②** Gut dysbiosis caused by a changed dietary pattern (e.g., high-grain diet-induced SARA) or antibiotic reduces polysaccharide-degrading commensal bacteria and prompts free SAs production by increasing sialidase, causing abundant free SAs in the intestinal lumen which fuels polysaccharide-degrading opportunistic pathogen (e.g., *Enterobacteriaceae* and *Moraxellaceae*). **③** Polysaccharide-degrading opportunistic pathogen, such as *Enterobacteriaceae*, proliferate by using SAs as an energy source, which facilitates their expansion and virulence enhancement, prompting the degradation of the mucus layer and gut barrier damage. **④** A leaky gut barrier allows toxic factors (e.g., LPS) to enter intestinal tissues and induces immune cell activation through TLR4-NF-κB/NLRP3 signatures, causing intestinal inflammation. **⑤** The increased LPS derived from expansion of gut opportunistic pathogen subsequently enters the blood and, together with proinflammatory cytokines, induces systemic inflammation. **⑥** Persistent chronic systemic low-grade inflammation disrupts the blood-milk barrier, causing cytokines and LPS to enter and accumulate in the mammary gland, inducing mastitis through TLR4-NF-κB/NLRP3 pathways

Collectively, the proposed mechanisms by which SAs facilitate mastitis are summarized in Fig. 12. There are few free SAs in the intestinal lumen during homeostasis and the released free SAs are degraded by commensal bacteria. Gut dysbiosis resulting from many factors, such as SARA or antibiotics [34], increases the release of SAs. In addition, the reductions in polysaccharide-degrading commensal microbes caused by gut dysbiosis facilitate the accumulation of free SAs and the growth of facultative anaerobic opportunistic pathogen. Abundant free SAs are next degraded by polysaccharide-degrading opportunistic pathogen to prompt their expansion and virulence enhancement, leading to aggravated gut dysbiosis and increased production of LPS from opportunistic pathogen. Increased LPS and opportunistic pathogen disrupt gut barrier integrity and induce immune cell activation via the TLR4-NF-κB/NLRP3 pathways, prompting intestinal inflammation. Increased gut barrier permeability allows increased LPS and proinflammatory cytokines to translocate into systemic circulation, triggering systemic inflammation. Increased LPS and systemic inflammation subsequently disrupt the blood-milk barrier [49, 63] and facilitate the development of mastitis through TLR4-NF-κB/NLRP3 signatures.

## Conclusions

The current study revealed marked metabolic changes in SARA cows and identified that increased SA metabolism facilitated the development of gut dysbiosis-induced mastitis in mice. The underlying mechanism is involved in the overexpansion of *Enterobacteriaceae* and subsequent gut barrier damage and systemic inflammation by TLR4-NF-κB/NLRP3 signatures (Fig. 12). In contrast, depletion of *Enterobacteriaceae*, treatment with commensal bacteria and reduction of free SAs improved gut dysbiosis-associated mastitis in mice. Our findings constitute the first demonstration, to our knowledge, that SARA-derived metabolic changes exacerbate systemic inflammation and mastitis by facilitating gut opportunistic pathogen expansion through the microbiota-gut-mammary axis. Likewise, in addition to highlighting the adverse impact of host-derived sugar on mastitis in the context of gut dysbiosis, the findings of this study also indicate that inhibition of SA release and maintenance of gut homeostasis might be an alternative strategy to control mastitis by blocking the gut-mammary axis in the context of gut dysbiosis.

## Material and methods

### Cows treatment and samples collection

Twelve Holstein cows (4–6 years, averaging ~ 600 kg of weight) were obtained from a farm in Qingzhou, Shangdong Province, China, and none of the animals had diseases or treated with antibiotics and drugs within 6 months. Cows separated into health groups and SARA group randomly. All cows meet the daily nutrient requirements for lactating. The health group was treated with a standard diet of grass-legume hay and SARA group was treated with a HGD (70% grain and 30 forage diet) as previous described [27, 83]. After 8 weeks treatment, the rumen fluid from different treated cows were harvested according to previous study [27]. Briefly, each ruminal sample was harvested by rumenocentesis using a nonpyrogenic needles (2.0 × 120 mm) and 50 mL syringes. All samples were stored at liquid nitrogen until metabolomics analysis. Rumen pH was used to diagnosis SARS as previously [29].

### Untargeted metabolomics

Rumen fluid (100 mL) was mixed with prechilled methanol (400 μL) by well vortexing and then incubated on ice for 5 min, followed by centrifuging at 15,000 rpm, 4 °C for 5 min. LC–MS grade water was used to dilute the supernatant to a final concentration containing 53% methanol. After transferring to a fresh Eppendorf tube, the samples were centrifuged at 15,000 × *g*, 4 °C for 10 min and injected into the LC–MS/MS system analysis.

LC–MS/MS analyses were performed using a Vanquish UHPLC system (Thermo Fisher) coupled with an Orbitrap Q Exactive series mass spectrometer (Thermo Fisher). Samples were injected onto a Hyperil Gold column (100 × 2.1 mm, 1.9 μm) using a 16-min linear gradient at a flow rate of 0.2 mL/min. The raw data files generated by UHPLC-MS/MS were processed using the Compound Discoverer 3.1 (CD3.1, Thermo Fisher) to perform peak alignment, peak picking, and quantitation for each metabolite. The main parameters were set as follows: retention time tolerance, 0.2 min; actual mass tolerance, 5 ppm; signal intensity tolerance, 30%; signal/noise ratio, 3; and minimum intensity, 100,000. After that, peak intensities were normalized to the total spectral intensity. The normalized data was used to predict the molecular formula based on additive ions, molecular ion peaks and fragment ions. And then, peaks were matched with the mzCloud (https://www.mzcloud.org/), mzVault and MassList database to obtain the accurate qualitative and relative quantitative results. Statistical analyses were performed using the statistical software R (R version R-3.4.3), Python (Python 2.7.6 version), and CentOS (CentOS release 6.6), When data were not normally distributed, normal transformations were attempted using of area normalization method.

These metabolites were annotated using the KEGG database (http://www.genome.jp/kegg/), HMDB database (http://www.hmdb.ca/) and Lipidmaps database (http://www.lipidmaps.org/). PCA and PLS‐DA were performed at metaX. Univariate analysis (*t* test) was applied to calculate the statistical significance (*P* value). The metabolites with VIP > 1 and *P* value < 0.05 and FC ≥ 2 or FC ≤ 0.5 were considered to be differential metabolites. Volcano plots were used to filter metabolites of interest which based on Log2 (FC) and -log10 (*P* value) of metabolites. For clustering heat maps, the data were normalized using z-scores of the intensity areas of differential metabolites and were ploted by Pheatmap package in R language. The functions of these metabolites and metabolic pathways were studied using the KEGG database. The metabolic pathway enrichment of differential metabolites were performed, when ratio were satisfied by x/n > y/N, metabolic pathway were considered as enrichment, when *P* value of metabolic pathway < 0.05, metabolic pathway were considered as statistically significant enrichment.

### Mouse husbandry condition and treatments

All SPF grade BABL/c mice (22–24 g) used in the present study were obtained from Liaoning Changsheng Biotecnology Co., Ltd. (Benxi, China). Mice were raised in SPF grade feeding conditions with 12-h light daily for a week. These mice were then mixed at a ratio of three females to one male in separated cages and the male mice were removed after confirming pregnancy by vaginal spermatozoa.

For the SA supplementation experiment, 14 days of pregnancy mice were separated into four groups randomly, referred to as (1) Control (C) group: feeding with standard diet (AIN93G) and free water and treated with 200 μL PBS orally twice a day for 21 days; (2) Neu5Ac (N) group: treating with 1% Neu5Ac (Sigma Aldrich, MO, USA) in the water and orally gavaged 1 mg/200 μL of Neu5Ac twice a day for 21 days [34]; (3) Ampicillin (A) group: mice were treated with 1 g/L ampicillin (Sigma Aldrich, MO, USA) for 21 days modified according to previous study [36] and orally gavaged with 200 μL PBS twice a day; (4) Ampicillin + Neu5Ac (AN) group: mice were treated with antibiotics for 21 days accompanied with 1% Neu5Ac treatment and orally gavaged 1 mg/200 μL of Neu5Ac twice a day as described above. At the day 14 after delivery, mice were sacrificed and feces, mammary gland, ileum, colon and liver were harvested and stored at − 80 ℃ until detection.

For sodium tungstate treatment experiment, 0.8% sodium tungstate (RHAWN, Shanghai, China) was added in drinking water along with ampicillin and Neu5Ac treatments [47, 72]. For *L. reuteri* supplementation experiment, mice were treated ampicillin and Neu5Ac for 14 days before parturition, and then supplemented with *L. reuteri* CNCM I-5022 (10^9^ CFU) daily for 14 days through oral gavage [22].

For FMT or RMT experiments, feces or ruminal samples from different treated groups were harvested and prepared as previously described [19, 20, 46, 48, 49]. Briefly, fresh feces from each group were pooled and homogenized using sterile saline (50 mg feces/mL) [64]. In addition, a total of 6 ruminal samples (0.5 g/sample) were mixed and dispensed according to the experimental demand. These samples were centrifuged at 100 × *g*, 4 ℃ for 2 min, and then the supernatant were collected. For the transplantation, the mice (pregnancy for a week) were treated with antibiotic (200 mg/kg ampicillin, metronidazole (Sigma-Aldrich, MO, USA) and neomycin (Sigma-Aldrich, MO, USA) and 100 mg/kg vancomycin (Sigma-Aldrich, MO, USA)) daily through oral gavage for 5 days to eliminate commensal microbiota [46]. Furthermore, the antibiotics were removed for 1 day and mice were colonized with ruminal microbiota. In data, mice were oral gavaged with 300 μL fecal or ruminal supernatant for three consecutive days after antibiotics removal, and once every 2 days thereafter for 19 days with prepared microbiota samples [46]. For RMT supplemented with a HSD, mice were fed with a high-starch (5% cellulose) diet during RMT based on previously described [48]. For the sialidase inhibition experiment, mice were performed RMT as mentioned above and treated with zanamivir (0.5 mg/mL in drinking water, Yuanyebio, China) for 21 days [33].

### Bacteria culture and growth assay

To investigate the effect of SA on bacterial growth, *E. coli* AIEC strain LF82 was incubated in M9 medium (Sigma-Aldrich, MO, USA) supplemented with 10 mM Neu5Ac or glucose and the bacteria intensities were assessed at 600 nm optical density (OD600) [34]. To investigate the influence of SA on the virulent gene expressions of *E. coli*, *E. coli* LF82 were incubated in M9 medium with 2.5, 5, or 10 mM Neu5Ac or glucose, and then the virulent genes were determined using qPCR [45]. *L. reuteri* CNCM I-5022 was grown in in MRS (Haibo, Qingdao, China) as previously described [22].

### Cell culture and inflammatory responses assay

Macrophages (RAW264.7) were performed to confirm the effect of SA primed *E. coli* on inflammatory responses. Macrophages were cultured in 1640 medium (Sigma-Aldrich, MO, USA) suppled with 10% fetal bovine serum (BI, Israel) and 1% ampicillin and streptomycin at 37 ℃ with 5% CO_2._ For the *E. coli* stimulation experiment, cells (10^6^ cells/mL) were incubated in 24-well plates and removed the ampicillin and streptomycin (Sigma Aldrich, MO, USA), and then cells were treated with Neu5Ac or glucose-cultured *E. coli* (10^6^, 10^7^, and 10^8^ CFU) for 24 h. Finally, the supernatants were harvested and proinflammatory cytokines were determined by ELISA.

### Histological analysis

All samples used for histological analysis, including mammary glands, ileums, colons, and livers, were fixed with 4% paraformaldehyde for 48 h and embedded in paraffin to prepare 5-μm paraffin sections. All the sections were stained with hematoxylin and eosin (H&E) after dewaxing and hydration. The histological changes of different tissues were performed by blinded pathologists using an optical microscope (Olympus, Tokyo, Japan) and the histological scores were assessed as previously described [22].

### Cytokines assay

ELISA assay was performed to determine the predominant proinflammatory cytokines of TNF-α and IL-1β in the serums, mammary glands and cultured cell supernatants. For the mammary samples, 10% tissue homogenates were prepared using PBS and supernatants were collected by centrifuging at 12,000 rpm for 10 min. The concentrations of TNF-α and IL-1β in serums, mammary glands and cell supernatants were assessed and calculated according to the manufacturer’s instructions (Biolegend, CA, USA).

### MPO activity, ALT, and AST assay

To determine the MPO levels, 10% tissue homogenates were prepared using MPO buffer and detected by MPO assay kit according to the manufacturer’s instructions (Nanjing Jiancheng Bioengineering Institute, Nanjing, China). To investigate liver injury, the serum ALT and AST were determined using ALT and AST assay kits according to the manufacturer’s instructions (Nanjing Jiancheng Bioengineering Institute, Nanjing, China).

### LPS concentrations determination

LPS assay kit was performed to measure the LPS level in the serum or ruminal samples according to the manufacturer’s instructions (Lanpaibio, Shanghai, China). In brief, blood were collected and centrifuged at 3000 rpm for 10 min to separate the serum carefully. Rumen fluids were collected by centrifuge without heat source, precipitation was removed by centrifuging at 3000 rpm for 10 min, and supernatants were harvested for LPS determination.

### Lipocalin-2 determination

The concentrations of fecal lipocalin-2 were detected using lipocalin-2 ELISA kit according to manufacturer’s instructions (Lanpaibio, Shanghai, China).

### Sialidase activity and sialic acid assays

Sialidase activity in ruminal and intestinal samples and intestinal Neu5Ac levels were assessed by using the Sialidase Activity Assay Kit (MAK121, Sigma-Aldrich, MO, USA) and the Sialic Acid Assay Kit (MAK314, Sigma-Aldrich, MO, USA) following the manufacturer’s protocols, respectively.

### Goblet assessment by AB-PAS staining

The goblet cell and intestinal morphological analysis were performed by AB-PAS staining (Solarbio, Beijing, China) as previous described [40]. In brief, paraffin sections were dewaxed washed with water for 2 min, and then stained with AB for 15 min followed by washing three times per 2 min with water. Furthermore, the sections were oxidized using oxidant and washed for twice, and then the sections were stained by Schiff regent for 15 min followed by washing for 10 min. The nuclear was stained by hematoxylin solution for 2 min. Finally, the sections were treated by Scott Bluing Solution for 3 min and washed for 3 min. After dehydrating by series of ethanol and transparent by xylene, the sections were sealed with resinene and determined by blinded pathologists using an optical microscope (Olympus, Tokyo, Japan).

### Immunochemistry

Immunochemistry staining was performed to measure the Mucin 2 expression in the colon using SAP (Mouse/Rabbit) IHC Kit (MXB, China) as previous described [40]. In brief, colon paraffin sections were dewaxed by xylene and series of alcohol. The prepared sections were subjected to for antigen retrieval using sodium citrate following phosphate buffer (PBS) wash. Prepared sections were treated with endogenous peroxidase blockers for 40 min at room temperature. After washing by PBS for 3 times per 5 min, the sections were treated with normal nonimmune goat serum for 40 min at room temperature and then incubated with mucin-2 antibody (1:200, diluted with 5% goat serum) overnight at 4 °C. Furthermore, the sections were incubated with the secondary antibody (goat-anti rabbit IgG) for 30 min at room temperature after being washed with PBS. The sections were then incubated with horseradish peroxidase for 20 min at room temperature. After being washed with PBS, the sections were developed for 5 min using a color developing agent under the microscope and terminated by water. The nuclei were staining with hematoxylin for 2 min followed by 1% muriatic acid alcohol differentiation and ammonium hydroxide treatment. Following dehydration, the sections were mounted utilizing neutral resins and determined by blinded pathologists using an optical microscope (Olympus, Tokyo, Japan).

### Total bacterial DNA extraction and Illumina MiSeq sequencing

Microbial community genomic DNA was extracted from mouse feces or ruminal samples using the FastDNA® Spin Kit for Soil (MP Biomedicals, USA) according to manufacturer’s instructions. The DNA extract was checked on 1% agarose gel, and DNA concentration and purity were determined with NanoDrop 2000 UV–vis spectrophotometer (Thermo Scientific, Wilmington, USA). The hypervariable region V3-V4 of the bacterial 16S rRNA gene were amplified with primer pairs 338F (5′-ACTCCTACGGGAGGCAGCAG-3′) and 806R (5′-GGACTACHVGGGTWTCTAAT-3′) by an ABI GeneAmp® 9700 PCR thermocycler (ABI, CA, USA). The PCR amplification of 16S rRNA gene was performed as follows: initial denaturation at 95 ℃ for 3 min, followed by 27 cycles of denaturing at 95 ℃ for 30 s, annealing at 55 ℃ for 30 s and extension at 72 ℃ for 45 s, and single extension at 72 ℃ for 10 min, and end at 4 ℃. PCR reactions were performed in triplicate and the PCR product was extracted from 2% agarose gel and purified using the AxyPrep DNA Gel Extraction Kit (Axygen Biosciences, Union City, CA, USA) according to manufacturer’s instructions and quantified using Quantus™ Fluorometer (Promega, USA). Purified amplicons were pooled in equimolar and paired-end sequenced on an Illumina MiSeq PE300 platform/NovaSeq PE250 platform (Illumina, San Diego, USA) according to the standard protocols by Majorbio Bio-Pharm Technology Co. Ltd. (Shanghai, China). OTUs with 97% similarity cutoff were clustered using UPARSE version 7.1, and chimeric sequences were identified and removed. The taxonomy of each OTU representative sequence was analyzed by RDP Classifier version 2.2 against the 16S rRNA database using confidence threshold of 0.7. PCoA based on ANOSIM was used to identify microbial structure and LEfSe was performed to identify bacterial taxa that were differentially enriched in different treatment groups. A Wilcoxon rank-sum test (FDR < 0.05) was performed to identify the differential bacterial taxa between the two groups.

### Total RNA extraction and quantitative RT-PCR

The total RNA of colon and mammary tissues were extracted by Trizol (Invitrogen, CA, USA) as our previously described [22]. Briefly, 100 mg tissues were extracted by 1 mL Trizol and subjected to chloroform, isopropanol and 75% ethyl alcohol treatment under RNase-free conditions. After reverse transcription using TransStart Tip Green qPCR SuperMix (TransGen Biotech, Beijing, China), the cDNA was reacted with specific primers using a FastStart Universal SYBR Green Master Mix (ROX) (Roche, Switzerland, Basel) in a Step One Plus apparatus (Applied Biosystems, Foster City, CA, USA). The reaction conditions were performed as previously mentioned [22]. Primers used in this study are presented in Table S4, and GAPDH or rpoA served as an endogenous control for tissues or *E. coli*, respectively. The 2^−ΔΔCt^ method was performed to calculate the relative expression of genes through calibrating by the control group.

### Western blotting

The total protein of colon and mammary gland were harvested using a tissue protein extract (Thermo Fisher Scientific, USA) as previously described [22]. 10% or 15% SDS-PAGE were used to separate proteins and then combined with 0.45 μm PVDF membranes. After being blocked in 5% skim milk, the prepared PVDF membranes were incubated at 4 ℃ overnight with specific primary antibodies, including ZO-1, Occludin, Claudin-3, p-p65, p65, p-IκB, and IκB obtained from Affinity Biosciences (OH, USA), and TLR4, NLRP3, ASC, IL-1β and β-actin obtained from Cell Signaling Technology (CST, Boston, USA). After washing three times with TBST, the PVDF membranes were treated with Goat anti-rabbit or Rabbit anti-mouse IgG (1:20,000, Affinity Biosciences, OH, USA) for 2 h at room temperature. Ultimately, the proteins were determined by the ECL plus western blotting Detection System (Tanon, China).

### Statistical analysis

All data were analyzed using GraphPad Prism 8 (San Diego, CA, USA) and expressed as the boxplot or the mean ± SD. Student’s *t* test was performed for the comparison of two groups. One-way analysis of variance (ANOVA) was performed for the comparison more than two groups, followed by Tukey test to determine the differences among groups. *p* < 0.05 indicated statistical significance. Other special analysis was stated in legends.

## Supplementary Information

**Additional file 1: Figure S1.** SARA induces mastitis in cows. Cows were treated with a standard or high-grain diet for two months and ruminal, serum, milk and mammary gland tissues were harvested for analysis. A. Ruminal PH at day 60 from Health and SARA cows (*n*=6). B-C. Ruminal and serum LPS levels were detected using ELISA (*n*=6). D. Somatic cell count was performed in Health and SARA cows (*n*=6). E. Representative images of H&E-stained sections from Health and SARA samples. Red arrows indicate leukocyte infiltration. Blue arrows show the structure injury of mammary gland. Black arrows indicate edema. F. Histological score based on H&E-stained sections (*n*=6). Mammary TNF-α (G) and IL-1β (H) from Health and SARA groups were measured by ELISA (*n*=6). Each dot represents an individual cow (A-D and F-H) and Student’s t test was performed (A-D and F-H). **p < 0.01, ***p < 0.001 indicate significance. **Figure S2.** Data quality checks. A. The Pearson correlation of ruminal QC samples. B-C. The PCA score plots for Health and SARA samples containing QC samples (*n*=6). QC, quality control; PCA, Principal component analysis. **Figure S3.** Classification and functional annotation of metabolites. A. KEGG pathway annotation for Health and SARA ruminal samples. B. HMDB classification annotation. C. Lipid maps annotation for Health and SARA groups. HMDB, Human Metabolome Database; KEGG, Kyoto Encyclopedia of Genes and Genomes. **Figure S4.** SARA induced ruminal metabolic changes. A. PLS-DA score plots for ruminal samples (*n*=6). B. Cross-validation plot with a permutation test repeated 200 times. The intercepts of R2 (0.0, 0.57) and Q2 (0.0,–1.09) indicate that the PLS-DA model was not overfitting. C. Pathway enrichment analysis of significantly elevated metabolites in SARA sample according to the KEGG pathway. **Figure S5.** Spearman correlation between metabolites and inflammatory parameters. The red color denotes a positive correlation, while green color denotes a negative correlation. The intensity of the color is proportional to the strength of Spearman correlation. *p < 0.05, **p < 0.01, ***p < 0.001 indicate significance. **Figure S6.** Spearman correlation among metabolites. The top 20 correlated metabolites were showed based on the *P* value. The red color denotes a positive correlation, while blue color denotes a negative correlation. **Figure S7.** SA and FMT change the intestinal SA levels. A-B. The intestinal SA levels from different treatment groups (*n*=6-8). Data are expressed as the mean ± SD (A-B) and one-way ANOVA was performed, followed by Tukey test (A-B). *p < 0.05, **p < 0.01, ***p < 0.001 indicate significant difference. **Figure S8.** Sialic acid treatment has minimum effects on the synthesis of milk proteins and ileum histology. A-D. Relative mammary gene expressions associated with the synthesis of milk proteins from indicated groups, including Csn1, Csn2, Csn3, and Wap (*n*=7-8). E. The average weight changes of litters from different treatment groups (*n*=6). F. Representative images of H&E-stained ileum sections from indicated mice. G. Histological score based on H&E-stained sections (*n*=7-8). **Figure S9.** Composition of the gut microbiota in different groups. A. Chao1 index in different groups (*n*=7-8). B. Ace index (*n*=7-8). C. Bacterial composition at the family level in the gut were displayed (*n*=7-8). Each dot represents an individual mouse (A and B) and one-way ANOVA was performed, followed by Tukey test (A and B). ***p < 0.001 indicate significance. ns, no significance. **Figure S10.** Top 50 metabolism pathways enriched in different groups using Tax4Fun. **Figure S11.** Sodium tungstate treatment reduces intestinal Enterobacteriaceae abundance. A. The gut microbial compositions at the family level from different treatment groups (*n*=4). B. A Wilcoxon rank-sum test was performed to identify the differential bacterial taxa in the AN and AN+T groups (FDR < 0.05) (*n*=4). **Figure S12.** The ruminal and intestinal microbial compositions in the donor and recipient mice, and the effect of RMT on systemic inflammation in mice. A-D. Alpha diversity indices, including observed species (A), Shannon (B), Chao1 (C) and Simpson index (D), from Health and SARA groups (*n*=6). E. The ruminal microbial compositions at the family level from the indicated groups (*n*=6). F. Venn diagram showed the observed OTUs in different RMT groups. G-I. Alpha diversity indices, including Shannon (G), Chao1 (H) and Simpson index (I), from different treatment groups (*n*=7). J. The gut microbial compositions at the family level from different RMT groups (*n*=7). K-L. Serum TNF-α (K) and IL-1β (L) levels by ELISA (*n*=7). Data are expressed as boxplot (A-D and G-I) or the mean ± SD (K-L). A Student’s t test (A-D) or one-way ANOVA was performed, followed by Tukey test (G-I and K-L). *p < 0.05, **p < 0.01, ***p < 0.001 indicate significant difference. **Figure S13.** A high-starch diet promotes SA production and mastitis in SRMT mice. A. Neu5Ac levels from different treatment groups (*n*=5). B. Representative images of H&E-stained mammary sections. C. Histological scores based on H&E-stained mammary sections (*n*=5). D-F. Mammary TNF-α (D), IL-1β (E), MPO activity (F) were assessed (*n*=5). Data are expressed as the mean ± SD (C-F) and one-way ANOVA was performed, followed by Tukey test (C-F). **p < 0.01, ***p < 0.001 indicate significant difference. **Table S1.** Identified number of differential metabolites in Health and SARA samples. **Table S2.** Metabolites significantly upregulated in SARA cows and ranked according to the *P*-value. **Table S3.** Metabolites significantly downregulated in SARA cows and ranked according to the *P*-value. **Table S4.** The oligonucleotides used in this study.

## Data Availability

All data generated during the current study are included in this article (and its supplementary information files). The 16S rRNA gene sequencing data in the present study are available in NCBI Sequence Read Archive (SRA) repository under accession number PRJNA791422.

## Abbreviations

AB-PAS: Alcian blue-Periodic Acid Schiff
ALT: Alanine aminotransferase
ANOVA: One-way analysis of variance
AST: Aspartate aminotransferase
*E. coli*: *Escherichia coli*
FC: Fold change
FMT: Fecal microbiota transplantation
H&E: Hematoxylin and eosin
HMDB: Human metabolome database
HRMT: Ruminal microbiota transplantation from healthy cows
IL-1β: Interleukin-1β
KEGG: Kyoto Encyclopedia of Genes and Genomes
*L. reuteri*: *Lactobacillus reuteri*
LPS: Lipopolysaccharide
MPO: Myeloperoxidase
Neu5Ac: N-acetylneuraminic acid
Neu5Gc: N-glycolylneuraminic acid
NF-κB: Nuclear factor-κ-gene binding
NLRP3: NACHT LRR and PYD domains-containing protein 3
OTUs: Operational taxonomic units
PCA: Principal components analysis
PCoA: Principal coordinate analysis
PLS-DA: Partial least squares discriminant analysis
QC: Quality control
RMT: Ruminal microbiota transplantation
SARA: Subacute ruminal acidosis
*S. aureus*: *Staphylococcus aureus*
SCC: Somatic cell count
SRMT: Ruminal microbiota transplantation from cows with SARA-associated mastitis
SPF: Specific pathogen free
TLR4: Toll-like receptor 4
TJ: Tight junction
TNF-α: Tumor necrosis factor-α
VIP: Variable importance in the projection
