# Supplementary material for: Sialic acid exacerbates gut dysbiosis-associated mastitis through the microbiota-gut-mammary axis by fueling gut microbiota disruption
Source: Microbiome. 2023 Apr 17;11:78. doi: 10.1186/s40168-023-01528-8 (PMC10107595; doi:10.1186/s40168-023-01528-8)
Supplement: Supplementary file 2 — Additional file 1: Figure S1. SARA induces mastitis in cows. Cows were treated with a standard or high-grain diet for two months and ruminal, serum, milk and mammary gland tissues were harvested for analysis. A. Ruminal PH at day 60 from Health and SARA cows (n=6). B-C. Ruminal and serum LPS levels were detected using ELISA (n=6). D. Somatic cell count was performed in Health and SARA cows (n=6). E. Representative images of H&E-stained sections from Health and SARA samples. Red arrows indicate leukocyte infiltration. Blue arrows show the structure injury of mammary gland. Black arrows indicate edema. F. Histological score based on H&E-stained sections (n=6). Mammary TNF-α (G) and IL-1β (H) from Health and SARA groups were measured by ELISA (n=6). Each dot represents an individual cow (A-D and F-H) and Student’s t test was performed (A-D and F-H). **p < 0.01, ***p < 0.001 indicate significance. Figure S2. Data quality checks. A. The Pearson correlation of ruminal QC samples. B-C. The PCA score plots for Health and SARA samples containing QC samples (n=6). QC, quality control; PCA, Principal component analysis. Figure S3. Classification and functional annotation of metabolites. A. KEGG pathway annotation for Health and SARA ruminal samples. B. HMDB classification annotation. C. Lipid maps annotation for Health and SARA groups. HMDB, Human Metabolome Database; KEGG, Kyoto Encyclopedia of Genes and Genomes. Figure S4. SARA induced ruminal metabolic changes. A. PLS-DA score plots for ruminal samples (n=6). B. Cross-validation plot with a permutation test repeated 200 times. The intercepts of R2 (0.0, 0.57) and Q2 (0.0,–1.09) indicate that the PLS-DA model was not overfitting. C. Pathway enrichment analysis of significantly elevated metabolites in SARA sample according to the KEGG pathway. Figure S5. Spearman correlation between metabolites and inflammatory parameters. The red color denotes a positive correlation, while green color denotes a negative correlation. The inte [file 40168_2023_1528_MOESM1_ESM.docx]

**Supplementary materials for**

**Sialic acid exacerbates gut dysbiosis-associated mastitis through the microbiota-gut-mammary axis by fueling gut microbiota disruption**

Caijun Zhao^1#^, Xiaoyu Hu^1#^, Min Qiu^1^, Lijuan Bao^1^, Keyi Wu^1^, Xiangyue Meng^2^, Yihong Zhao^1^, Lianjun Feng^1^, Shiyu Duan^1^, Yuhong He^1^, Naisheng Zhang^1^*, Yunhe Fu^1^*

1. Department of Clinical Veterinary Medicine, College of Veterinary Medicine, Jilin University, Changchun 130062, Jilin Province, China.

2. Department of Breast Center, West China Hospital, Sichuan University, Chengdu 610041, Sichuan Province, China.

**#** Authors contribute equally to the present study

*** Corresponding author:**

Yunhe Fu, E-mail: fuyunhesky@163.com.

Naisheng Zhang, E-mail: zhangns@jlu.edu.cn.


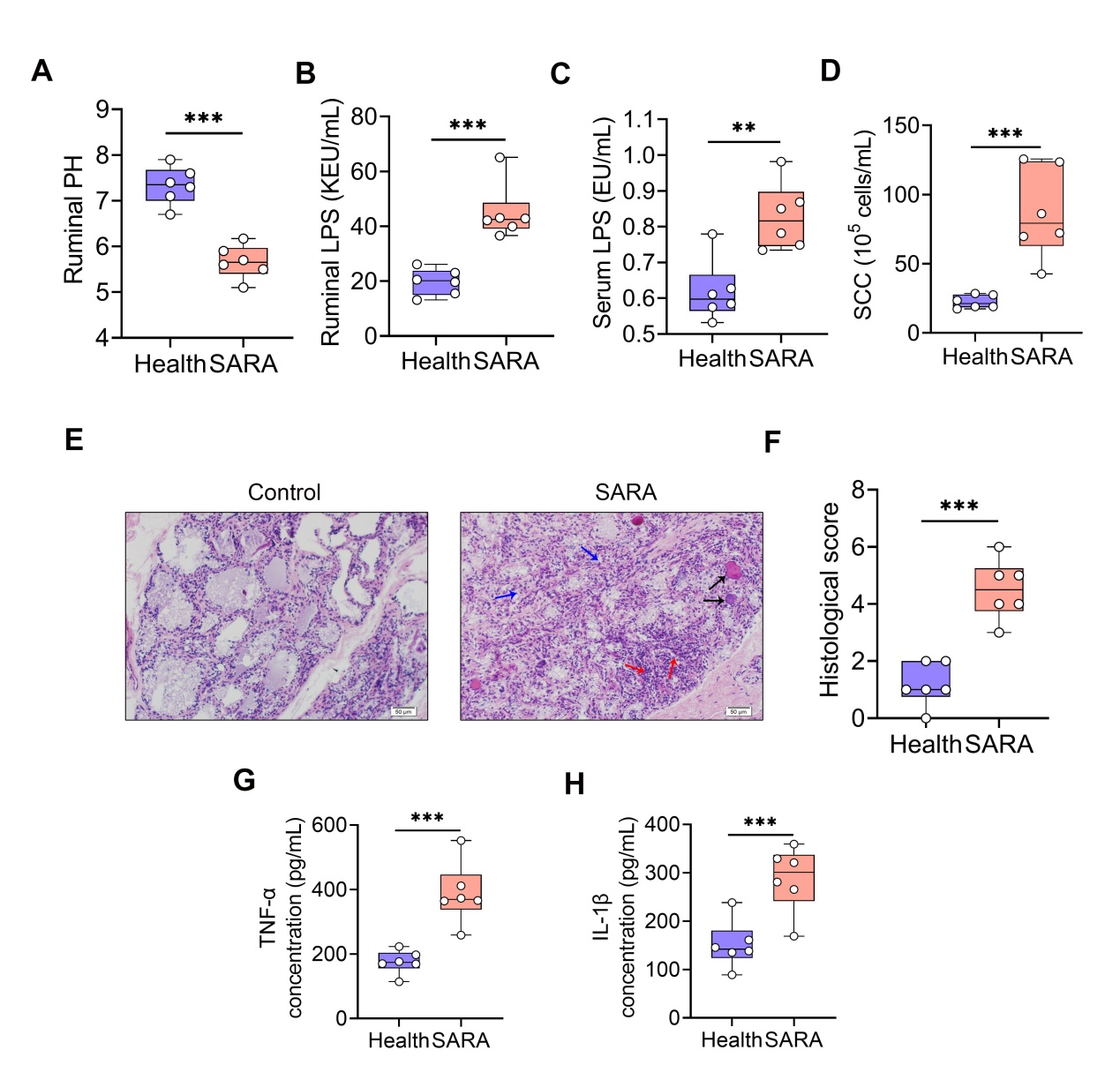


Fig. **S1 SARA induces mastitis in cows.** Cows were treated with a standard or high-grain diet for two months and ruminal, serum, milk and mammary gland tissues were harvested for analysis. **A.** Ruminal PH at day 60 from Health and SARA cows (n=6). **B**-C. Ruminal and serum LPS levels were detected using ELISA (n=6). **D.** Somatic cell count was performed in Health and SARA cows (n=6). **E**. Representative images of H&E-stained sections from Health and SARA samples. Red arrows indicate leukocyte infiltration. Blue arrows show the structure injury of mammary gland. Black arrows indicate edema. **F**. Histological score based on H&E-stained sections (n=6). Mammary TNF-α (**G**) and IL-1β (**H**) from Health and SARA groups were measured by ELISA (n=6). Each dot represents an individual cow (**A-D** and **F-H**) and Student’s t test was performed (**A-D** and **F-H**). ***p* < 0.01, ****p* < 0.001 indicate significance.


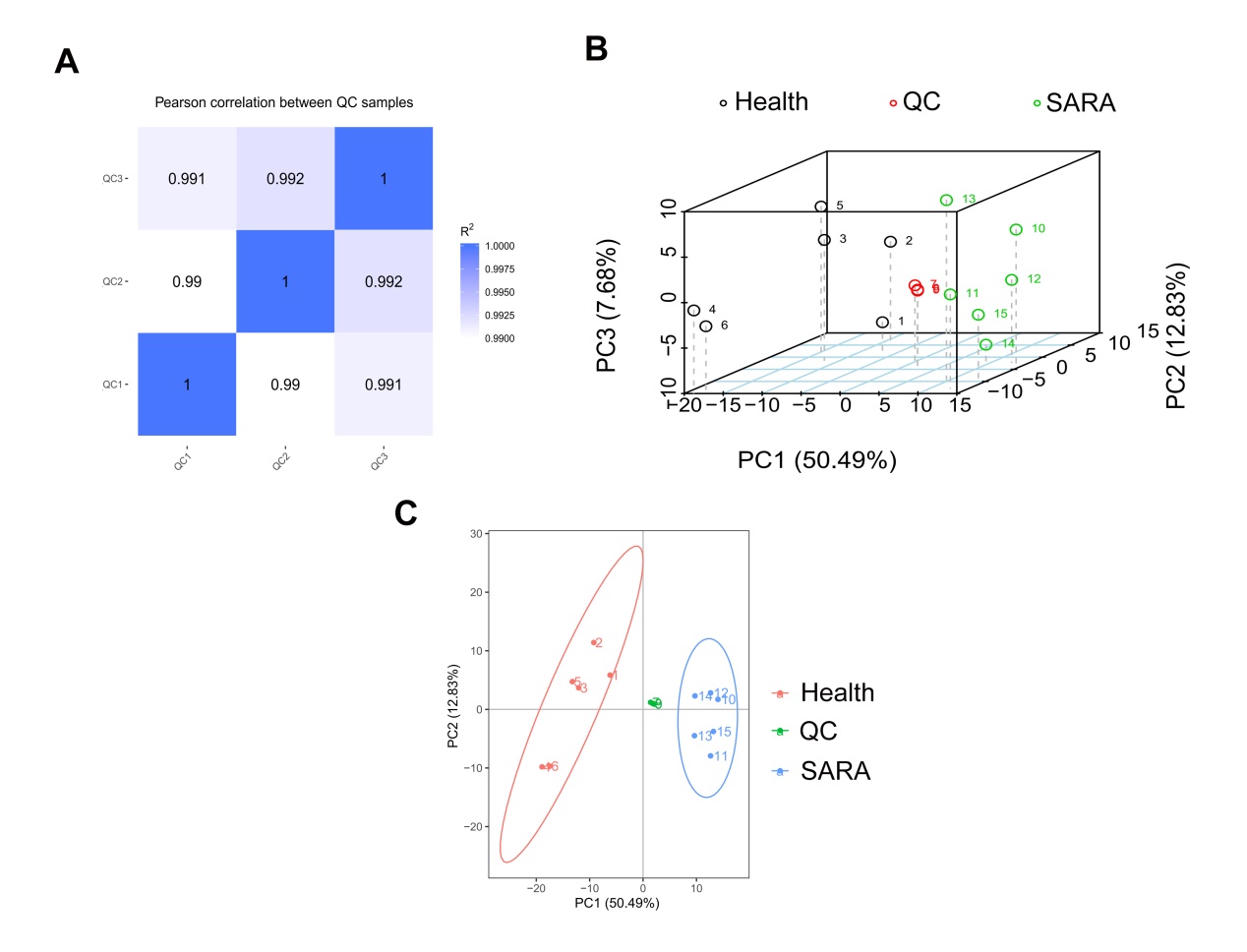


Fig. S2 **Data quality checks.** A. The Pearson correlation of ruminal QC samples. **B-C**. The PCA score plots for Health and SARA samples containing QC samples (n=6). QC, quality control; PCA, Principal component analysis.


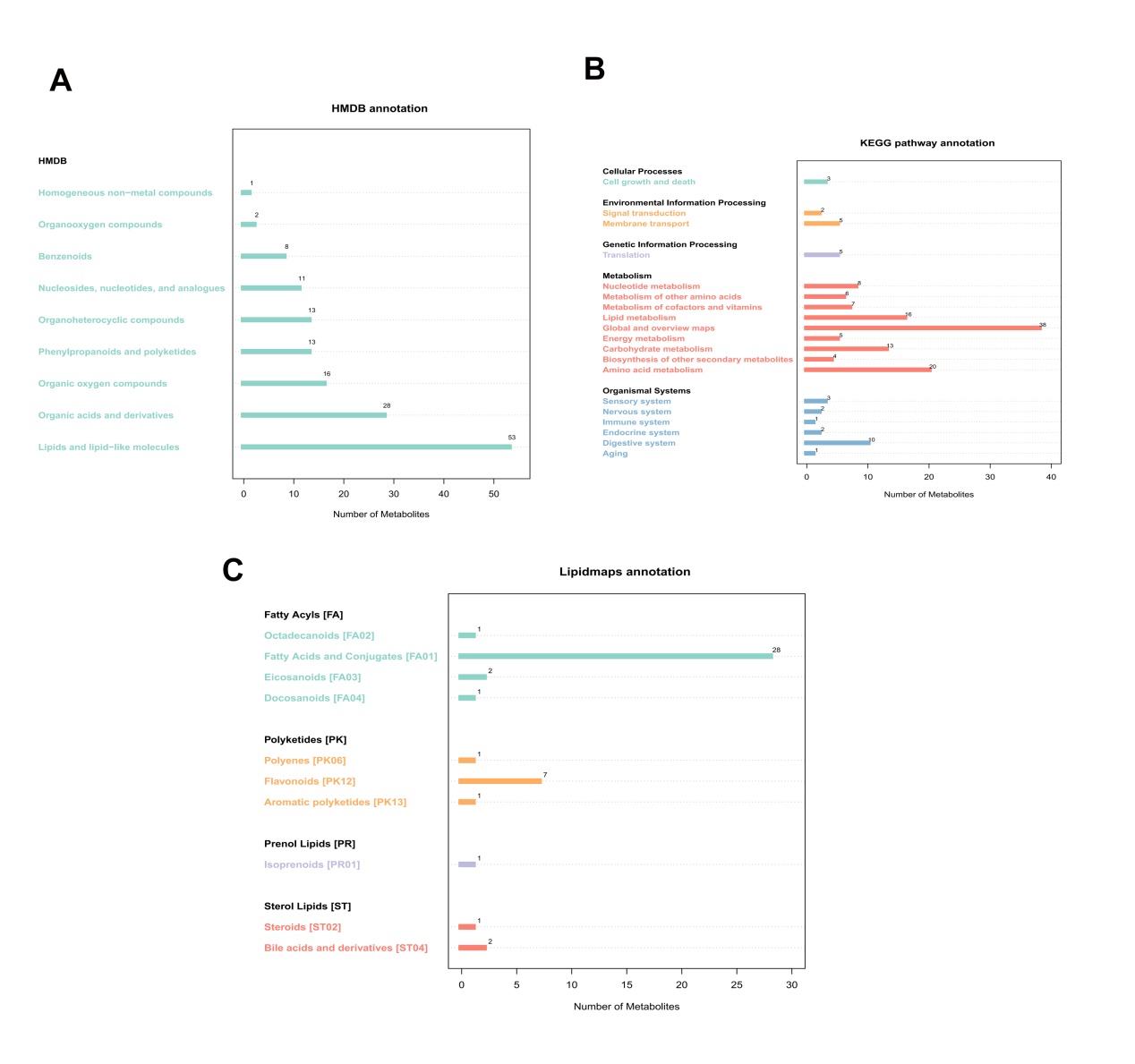


Fig. **S3 Classification and functional annotation of metabolites. A.** KEGG pathway annotation for Health and SARA ruminal samples. **B**. HMDB classification annotation. **C**. Lipid maps annotation for Health and SARA groups. HMDB, Human Metabolome Database; KEGG, Kyoto Encyclopedia of Genes and Genomes.


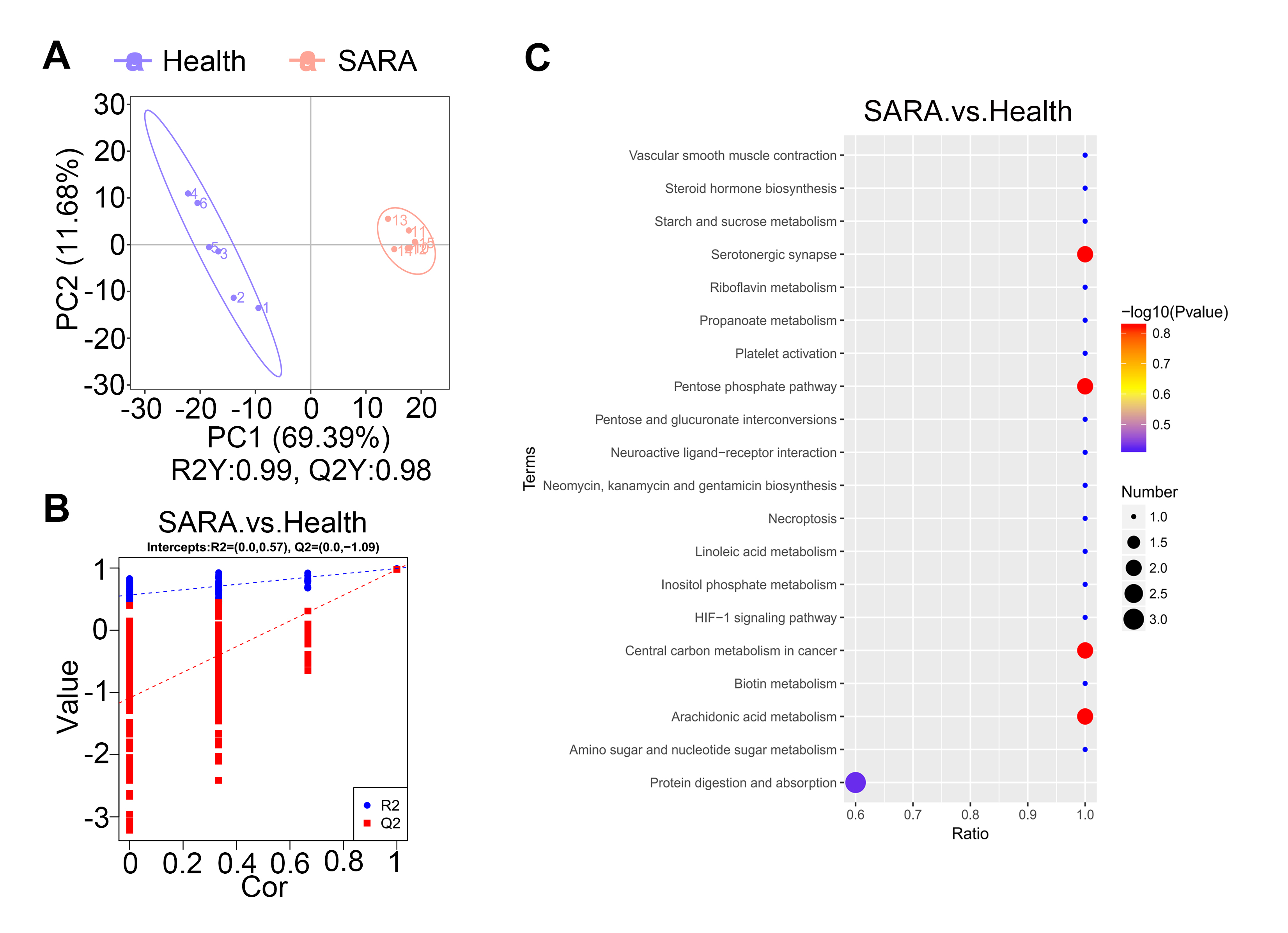


Fig. **S4 SARA induced ruminal metabolic changes. A**. PLS-DA score plots for ruminal samples (n=6). **B**. Cross-validation plot with a permutation test repeated 200 times. The intercepts of R2 (0.0, 0.57) and Q2 (0.0,–1.09) indicate that the PLS-DA model was not overfitting. **C.** Pathway enrichment analysis of significantly elevated metabolites in SARA sample according to the KEGG pathway.


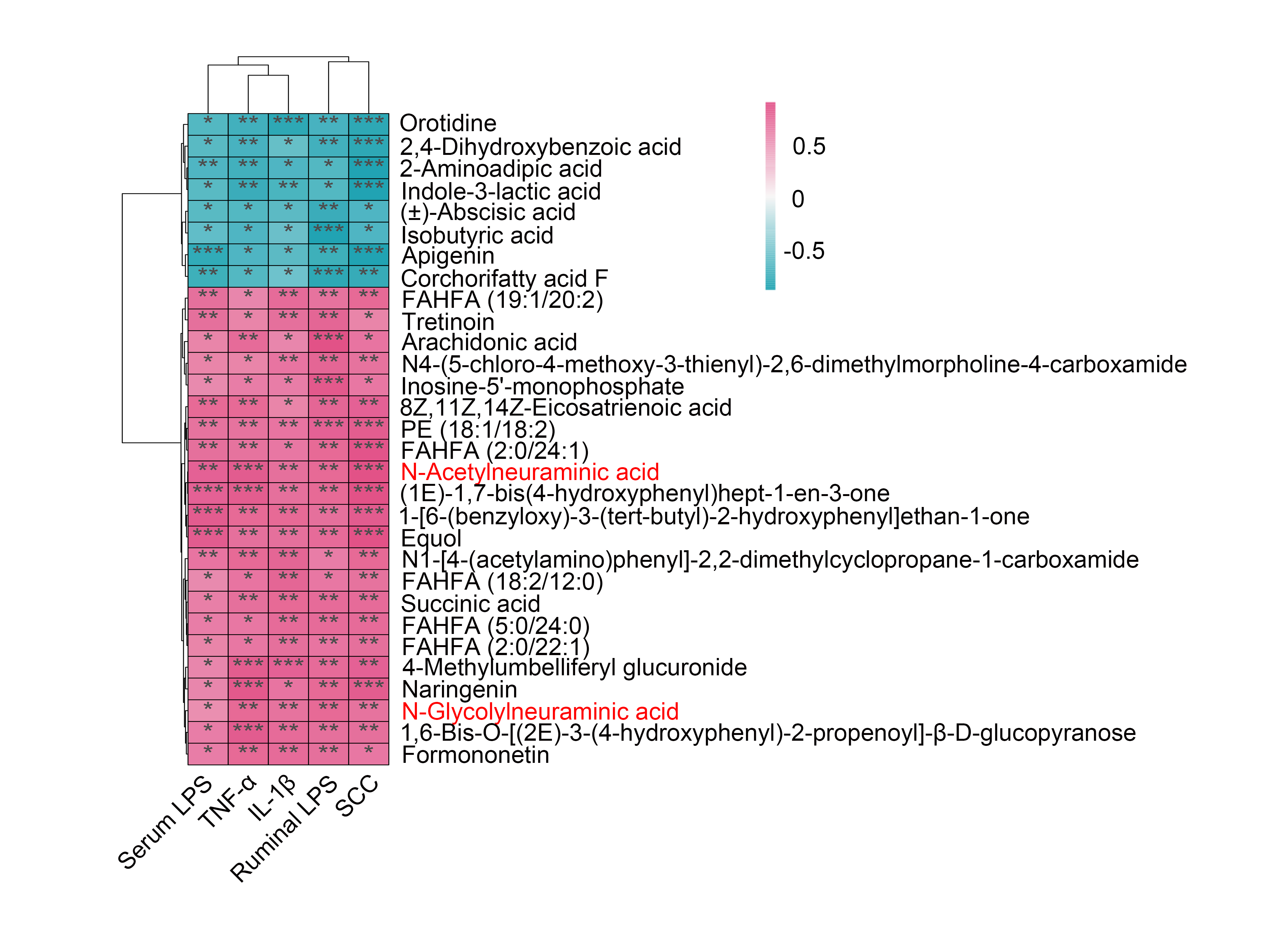


**Fig. S5** **Spearman correlation between metabolites and inflammatory parameters**. The red color denotes a positive correlation, while green color denotes a negative correlation. The intensity of the color is proportional to the strength of Spearman correlation. **p* < 0.05, ***p* < 0.01, ****p* < 0.001 indicate significance.


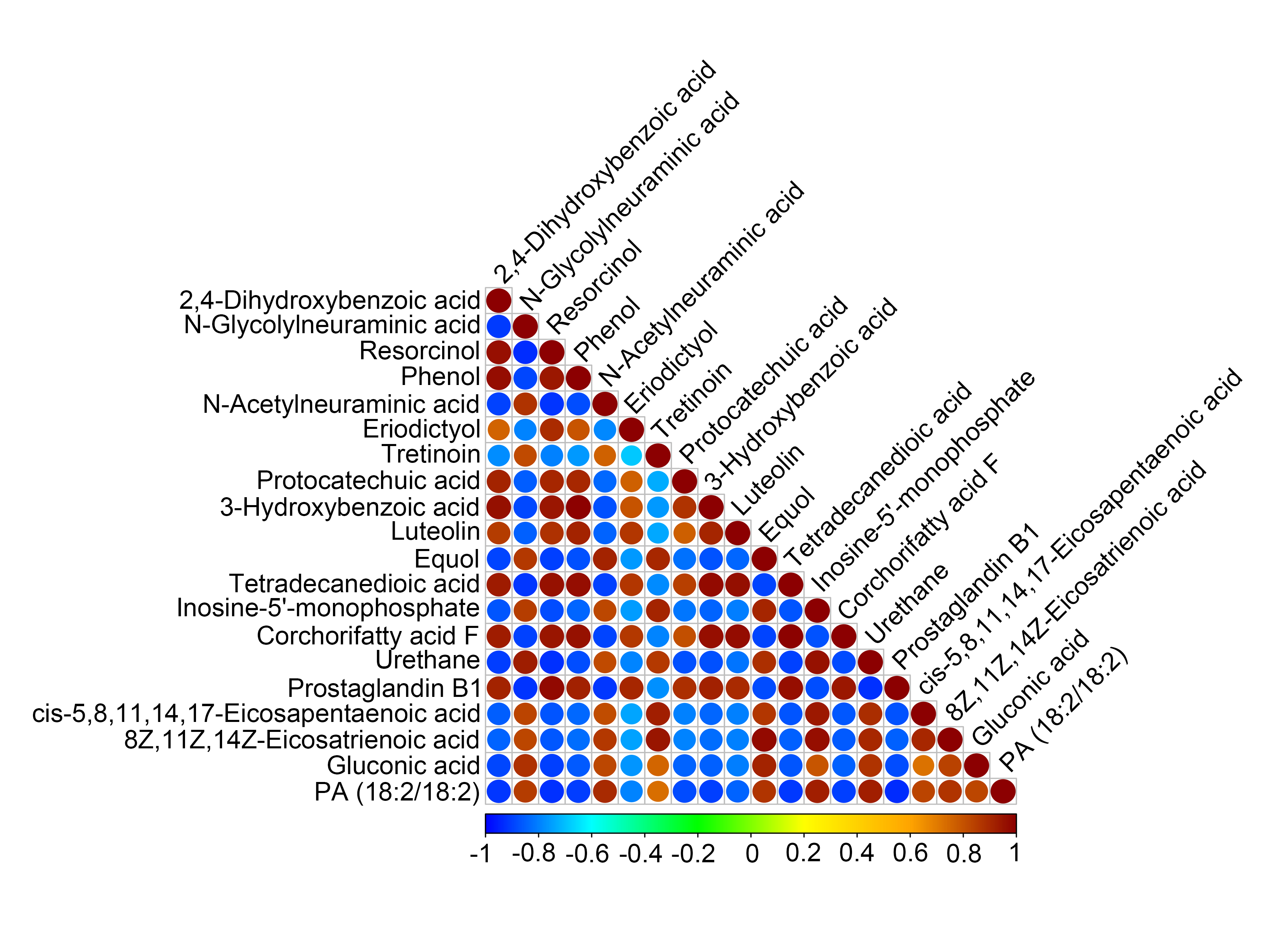


**Fig. S6 Spearman correlation among metabolites.** The top 20 correlated metabolites were showed based on the P value. The red color denotes a positive correlation, while blue color denotes a negative correlation.


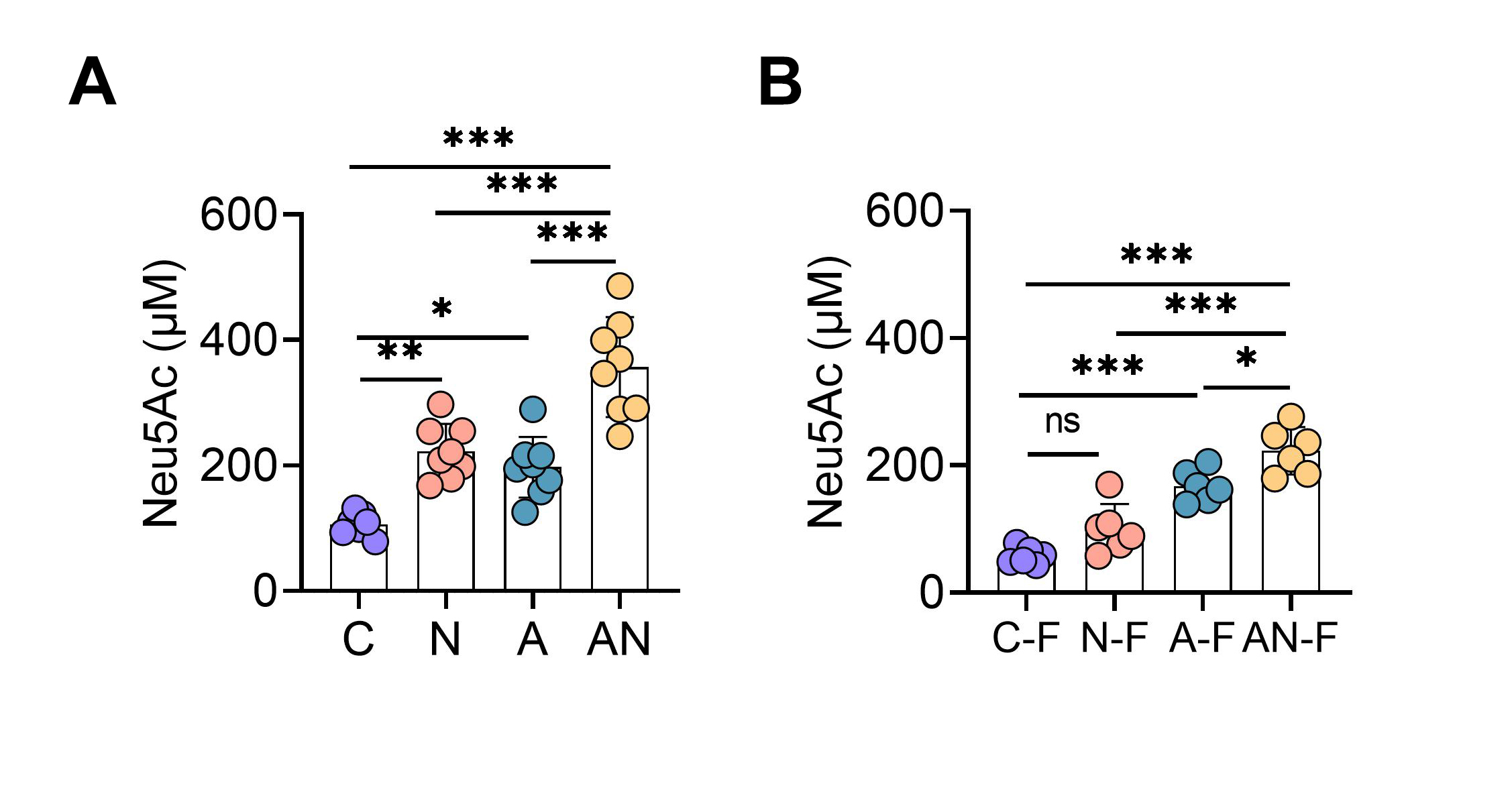


**Fig. S7 SA and FMT change the intestinal SA levels. A-B.** The intestinal SA levels from different treatment groups (n=6-8). Data are expressed as the mean ± SD (A-B) and one-way ANOVA was performed, followed by Tukey test (A-B). *p < 0.05, **p < 0.01, ***p < 0.001 indicate significant difference.


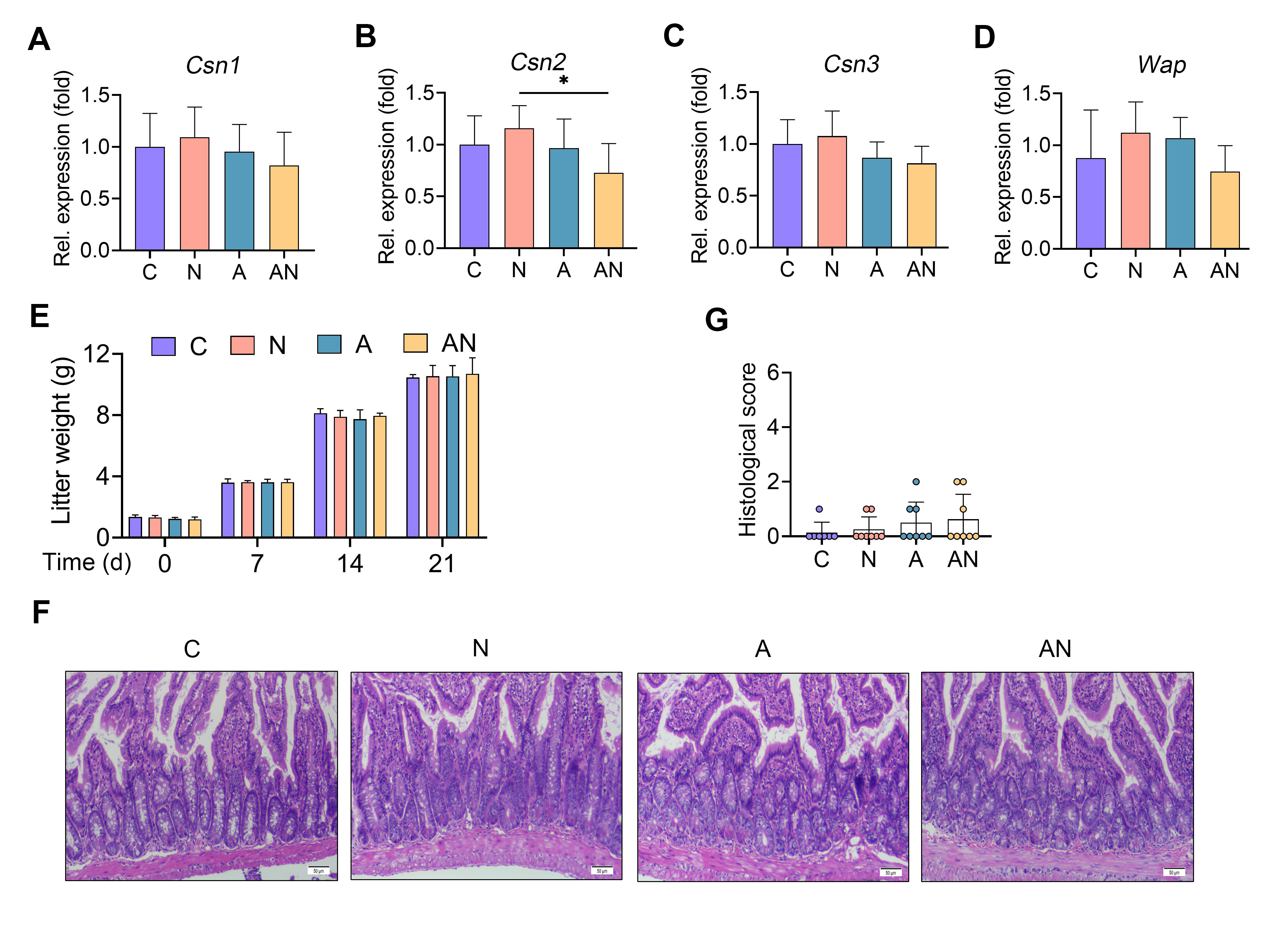


Fig. **S8 Sialic acid treatment has minimum effects on the synthesis of milk proteins and ileum histology. A-D.** Relative mammary gene expressions associated with the synthesis of milk proteins from indicated groups, including *Csn1*, *Csn2*, *Csn3*, and *Wap* (n=7-8). **E.** The average weight changes of litters from different treatment groups (n=6). **F.** Representative images of H&E-stained ileum sections from indicated mice. **G**. Histological score based on H&E-stained sections (n=7-8).


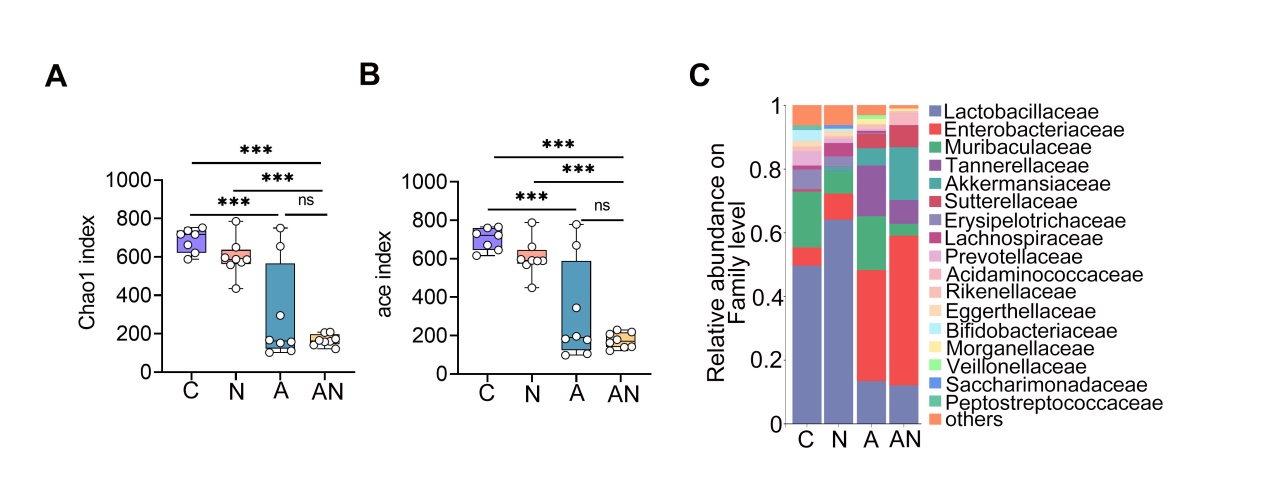


**Fig. S9 Composition of the gut microbiota in different groups. A.** Chao1 index in different groups (n=7-8). **B**. Ace index (n=7-8). **C.** Bacterial composition at the family level in the gut were displayed (n=7-8). Each dot represents an individual mouse (**A** and **B**) and one-way ANOVA was performed, followed by Tukey test (**A** and **B**). ****p* < 0.001 indicate significance. ns, no significance.


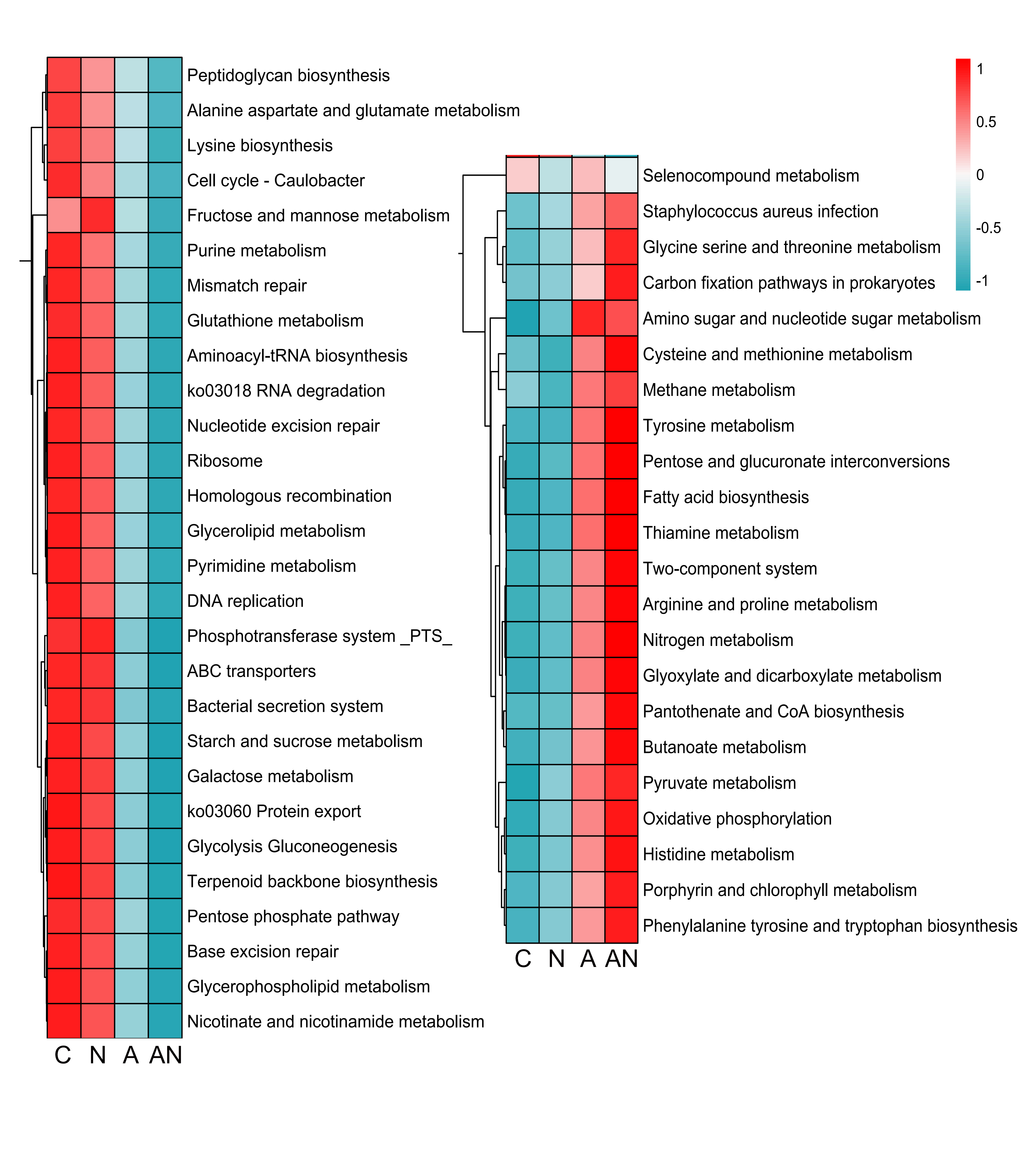


**Fig. S10 Top 50 metabolism pathways enriched in different groups using Tax4Fun.**

**
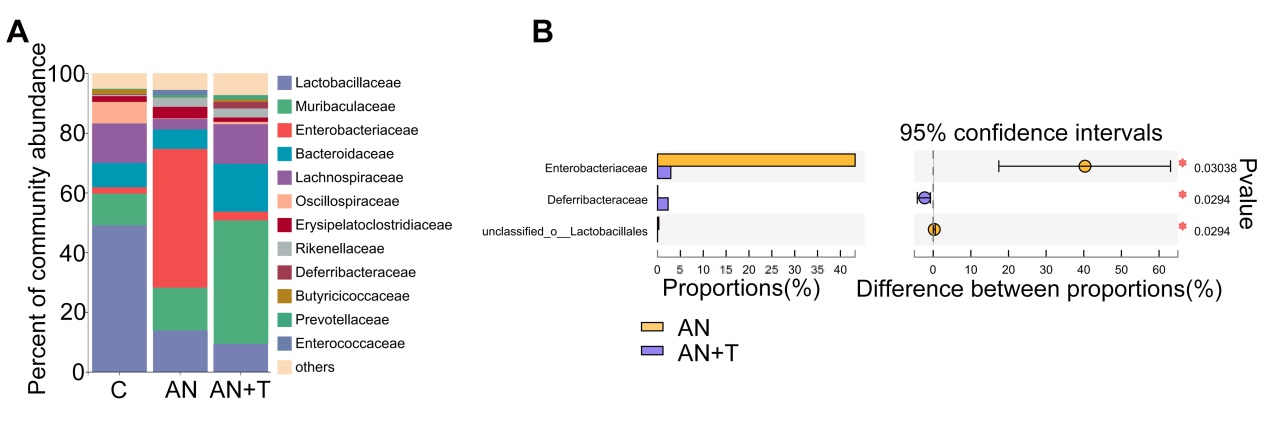
**

**Fig. S11 Sodium tungstate treatment reduces intestinal *Enterobacteriaceae* abundance**. **A**. The gut microbial compositions at the family level from different treatment groups (n=4). **B**. A Wilcoxon rank-sum test was performed to identify the differential bacterial taxa in the AN and AN+T groups (FDR < 0.05) (n=4).


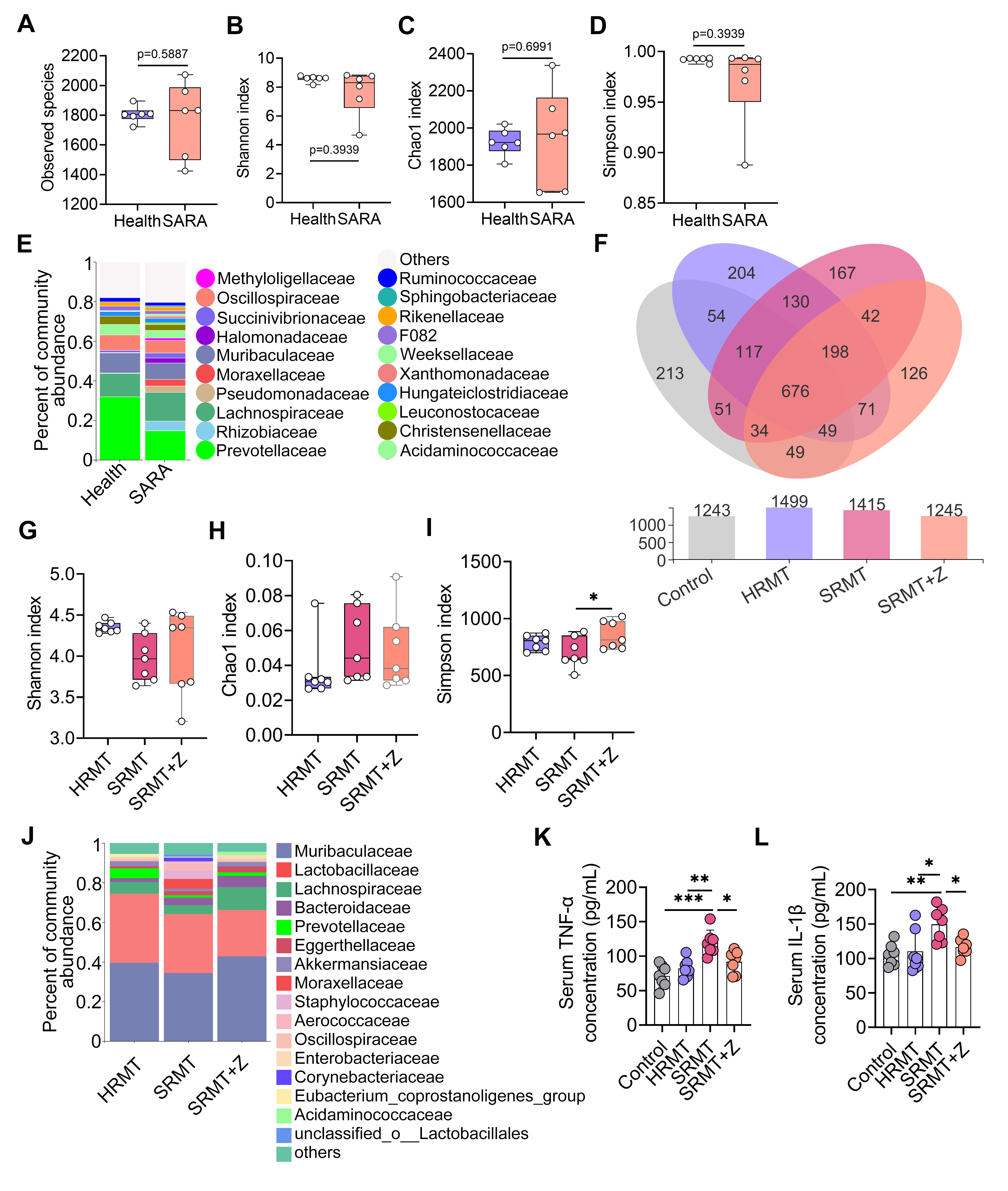


**Fig. S12 The ruminal and intestinal microbial compositions in the donor and recipient mice, and the effect of RMT on systemic inflammation in mice. A-D.** Alpha diversity indices, including observed species **(A)**, Shannon **(B)**, Chao1 **(C)** and Simpson index **(D)**, from Health and SARA groups (n=6). **E**. The ruminal microbial compositions at the family level from the indicated groups (n=6). **F.** Venn diagram showed the observed OTUs in different RMT groups. **G-I**. Alpha diversity indices, including Shannon **(G)**, Chao1 **(H)** and Simpson index **(I)**, from different treatment groups (n=7). **J**. The gut microbial compositions at the family level from different RMT groups (n=7). **K-L.** Serum TNF-α (**K**) and IL-1β (**L**) levels by ELISA (n=7). Data are expressed as boxplot (**A-D** and **G-I**) or the mean ± SD (**K-L**). A Student’s t test (**A-D**) or one-way ANOVA was performed, followed by Tukey test (**G-I** and **K-L**). *p < 0.05, **p < 0.01, ***p < 0.001 indicate significant difference.


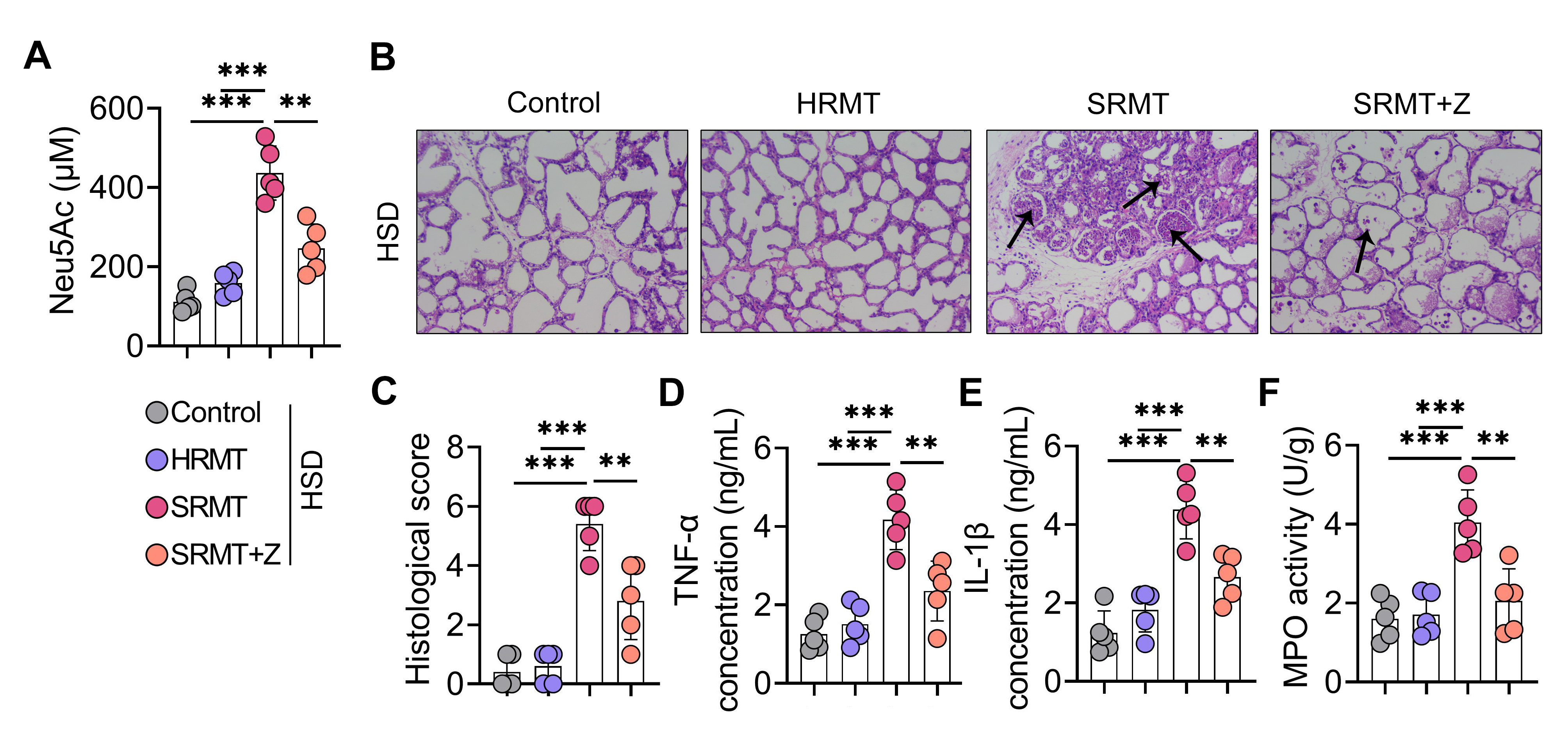


**Fig. S13 A high-starch diet promotes SA production and mastitis in SRMT mice. A.** Neu5Ac levels from different treatment groups (n=5). **B**. Representative images of H&E-stained mammary sections. **C**. Histological scores based on H&E-stained mammary sections (n=5). **D-F**. Mammary TNF-α (**D**), IL-1β (**E**), MPO activity (**F**) were assessed (n=5). Data are expressed as the mean ± SD (**C-F**) and one-way ANOVA was performed, followed by Tukey test (**C-F**). **p < 0.01, ***p < 0.001 indicate significant difference.

Table S1. Identified number of differential metabolites in Health and SARA samples.

| Compared Sample | Number of total identified | Number of total significant | Number of significant Up | Number of significant Down |
| --- | --- | --- | --- | --- |
| SARA. vs. Health | 282 | 83 | 49 | 34 |

**Table S2. Metabolites significantly upregulated in SARA cows and ranked according to the P-value**

| Name | Formula | FC | log2FC | P-value | VIP |
| --- | --- | --- | --- | --- | --- |
| N-Glycolylneuraminic acid | C11 H19 N O10 | 39.79765 | 5.314611 | 1.47E-09 | 2.618294 |
| N-Acetylneuraminic acid | C11 H19 N O9 | 16.16725 | 4.015003 | 8.52E-09 | 1.973257 |
| Tretinoin | C20 H28 O2 | 121.72 | 6.927422 | 1.50E-08 | 3.351196 |
| Equol | C15 H14 O3 | 4.453384 | 2.154902 | 2.89E-07 | 1.062092 |
| Inosine-5'-monophosphate (IMP) | C10 H13 N4 O8 P | 14.73215 | 3.880896 | 3.60E-07 | 1.894529 |
| Urethane | C3 H7 N O2 | 4.203734 | 2.071672 | 7.27E-07 | 1.018751 |
| cis-5,8,11,14,17-Eicosapentaenoic acid | C20 H30 O2 | 8.930898 | 3.158805 | 1.27E-06 | 1.539418 |
| 8Z,11Z,14Z-Eicosatrienoic acid | C20 H34 O2 | 25.77753 | 4.688042 | 1.40E-06 | 2.263389 |
| Gluconic acid | C6 H12 O7 | 9.188097 | 3.199766 | 1.51E-06 | 1.585949 |
| PA (18:2/18:2) | C39 H69 O8 P | 5.442327 | 2.444224 | 3.75E-06 | 1.21383 |
| Formononetin | C16 H12 O4 | 66.93374 | 6.064662 | 6.47E-06 | 2.822152 |
| FAHFA (2:0/24:1) | C26 H48 O4 | 4.919897 | 2.298628 | 1.37E-05 | 1.10321 |
| N1-[4-(acetylamino)phenyl]-2,2-dimethylcyclopropane-1-carboxamide | C14 H18 N2 O2 | 9.81104 | 3.294406 | 2.75E-05 | 1.578178 |
| 1-[6-(benzyloxy)-3-(tert-butyl)-2-hydroxyphenyl]ethan-1-one | C19 H22 O3 | 14.94665 | 3.90175 | 3.36E-05 | 1.846092 |
| PE (18:1/18:2) | C41 H76 N O8 P | 6.816542 | 2.76904 | 5.72E-05 | 1.311541 |
| 4-Methylumbelliferyl glucuronide | C16 H16 O9 | 14.15877 | 3.823624 | 6.26E-05 | 2.016826 |
| Succinic acid | C4 H6 O4 | 10.39446 | 3.377743 | 6.37E-05 | 1.728132 |
| (1E)-1,7-bis(4-hydroxyphenyl)hept-1-en-3-one | C19 H20 O3 | 8.81712 | 3.140308 | 7.12E-05 | 1.479328 |
| PE (18:2/18:2) | C41 H74 N O8 P | 5.422552 | 2.438972 | 7.15E-05 | 1.238694 |
| D-Glucose 6-phosphate | C6 H13 O9 P | 7.261463 | 2.86026 | 0.000191 | 1.338942 |
| Pimelic acid | C7 H12 O4 | 5.170669 | 2.370351 | 0.000245 | 1.110799 |
| 1,6-Bis-O-[(2E)-3-(4-hydroxyphenyl)-2-propenoyl]-β-D-glucopyranose | C24 H24 O10 | 4.252433 | 2.088288 | 0.000253 | 1.040014 |
| Pyrophosphate | H4 O7 P2 | 8.466053 | 3.08169 | 0.000598 | 1.673548 |
| Erucic acid | C22 H42 O2 | 5.977062 | 2.579437 | 0.000764 | 1.478566 |
| D-Ribulose 5-phosphate | C5 H11 O8 P | 5.643842 | 2.496678 | 0.0011 | 1.15481 |
| FAHFA (19:1/20:2) | C39 H70 O4 | 5.95077 | 2.573076 | 0.001139 | 1.429457 |
| Arachidonic acid | C20 H32 O2 | 6.413994 | 2.681223 | 0.001601 | 1.169733 |
| Nervonic acid | C24 H46 O2 | 6.679363 | 2.739711 | 0.002177 | 1.773861 |
| D-Sedoheptulose 7-phosphate | C7 H15 O10 P | 13.68504 | 3.774527 | 0.002195 | 1.624287 |
| PG (16:0/14:1) | C36 H69 O10 P | 3.879869 | 1.956008 | 0.002236 | 1.024176 |
| Adenosine5-phosphosulfate | C10 H15 N5 O10 P S | 14.18999 | 3.826802 | 0.002247 | 1.642932 |
| FAHFA (18:0/20:2) | C38 H70 O4 | 5.826751 | 2.542692 | 0.002586 | 1.660676 |
| Taurine | C2 H7 N O3 S | 45.90167 | 5.520475 | 0.003168 | 2.122659 |
| Naringenin | C15 H12 O5 | 4.385445 | 2.132723 | 0.003203 | 1.197322 |
| FAHFA (15:0/18:0) | C33 H64 O4 | 6.018517 | 2.589408 | 0.00334 | 1.305478 |
| FAHFA (18:2/12:0) | C30 H54 O4 | 3.441507 | 1.78304 | 0.003365 | 1.029775 |
| Arachidic acid | C20 H40 O2 | 5.103196 | 2.351401 | 0.00337 | 1.49028 |
| PE (16:0/18:2) | C39 H74 N O8 P | 3.290728 | 1.718407 | 0.004155 | 1.00119 |
| Heneicosanoic acid | C21 H42 O2 | 3.728673 | 1.898662 | 0.004205 | 1.145287 |
| all-cis-4,7,10,13,16-Docosapentaenoic acid | C22 H34 O2 | 7.519429 | 2.910623 | 0.005319 | 1.188745 |
| FAHFA (2:0/22:1) | C24 H44 O4 | 5.281424 | 2.400927 | 0.005501 | 1.639745 |
| N4-(5-chloro-4-methoxy-3-thienyl)-2,6-dimethylmorpholine-4-carboxamide | C12 H17 Cl N2 O3 S | 13.28307 | 3.731517 | 0.005915 | 1.547528 |
| FAHFA (5:0/24:0) | C29 H56 O4 | 3.928532 | 1.97399 | 0.006464 | 1.055527 |
| N'5-[3-(trifluoromethyl)benzoyl]-2,1,3-benzoxadiazole-5-carbohydrazide | C15 H9 F3 N4 O3 | 3.63951 | 1.863744 | 0.006504 | 1.118381 |
| FAHFA (14:0/18:1) | C32 H60 O4 | 3.350733 | 1.744477 | 0.008306 | 1.041973 |
| Ascorbic acid | C6 H8 O6 | 3.512602 | 1.81254 | 0.008376 | 1.206873 |
| FAHFA (16:0/18:0) | C34 H66 O4 | 4.470807 | 2.160535 | 0.01462 | 1.49034 |
| FAHFA (14:0/18:0) | C32 H62 O4 | 3.545851 | 1.826132 | 0.017622 | 1.248035 |
| FAHFA (16:0/18:1) | C34 H64 O4 | 3.33074 | 1.735843 | 0.025969 | 1.253426 |

FC, fold change; VIP, variable importance in the projection

**Table S3. Metabolites significantly downregulated in SARA cows and ranked according to the P-value**

| Name | Formula | FC | log2FC | P-value | VIP |
| --- | --- | --- | --- | --- | --- |
| 2,4-Dihydroxybenzoic acid | C7 H6 O4 | 0.035102 | -4.83232 | 3.17E-10 | 2.378124 |
| Resorcinol | C6 H6 O2 | 0.073384 | -3.7684 | 1.83E-09 | 1.857473 |
| Phenol | C6 H6 O | 0.087069 | -3.5217 | 8.02E-09 | 1.721819 |
| Eriodictyol | C15 H12 O6 | 0.002635 | -8.56794 | 1.46E-08 | 4.15838 |
| Protocatechuic acid | C7 H6 O4 | 0.020548 | -5.60487 | 4.02E-08 | 2.771074 |
| 3-Hydroxybenzoic acid | C7 H6 O3 | 0.061645 | -4.01988 | 1.40E-07 | 1.993561 |
| Luteolin | C15 H10 O6 | 0.035492 | -4.81637 | 1.54E-07 | 2.40542 |
| Tetradecanedioic acid | C14 H26 O4 | 0.121828 | -3.03708 | 3.20E-07 | 1.510479 |
| Corchorifatty acid F | C18 H32 O5 | 0.121067 | -3.04612 | 5.80E-07 | 1.517251 |
| Prostaglandin B1 | C20 H32 O4 | 0.197998 | -2.33644 | 1.06E-06 | 1.160279 |
| (±)-Abscisic acid | C15 H20 O4 | 0.160859 | -2.63613 | 4.11E-06 | 1.287745 |
| 3-Methoxyphenylacetic acid | C9 H10 O3 | 0.235807 | -2.08432 | 7.07E-06 | 1.008561 |
| Indole-3-lactic acid | C11 H11 N O3 | 0.171799 | -2.5412 | 9.82E-06 | 1.265735 |
| LPG 18:2 | C24 H45 O9 P | 0.127237 | -2.97441 | 1.88E-05 | 1.424025 |
| 18-β-Glycyrrhetinic acid | C30 H46 O4 | 0.063983 | -3.96618 | 2.36E-05 | 1.878223 |
| Hydrocinnamic acid | C9 H10 O2 | 0.229342 | -2.12443 | 6.45E-05 | 1.07746 |
| 3-[4-methyl-1-(2-methylpropanoyl)-3-oxocyclohexyl]butanoic acid | C15 H24 O4 | 0.223115 | -2.16414 | 6.88E-05 | 1.10373 |
| 3,8,9-trihydroxy-10-propyl-3,4,5,8,9,10-hexahydro-2H-oxecin-2-one | C12 H20 O5 | 0.215193 | -2.21629 | 0.000104 | 1.137385 |
| Isobutyric acid | C4 H8 O2 | 0.252124 | -1.98779 | 0.000114 | 1.002646 |
| diethyl 2-[(4-methoxy-2-nitroanilino)methylidene]malonate | C15 H18 N2 O7 | 0.167848 | -2.57478 | 0.000134 | 1.334107 |
| Apigenin | C15 H10 O5 | 0.169362 | -2.56182 | 0.000143 | 1.263201 |
| L-Histidine | C6 H9 N3 O2 | 0.20229 | -2.3055 | 0.000149 | 1.077415 |
| Orotidine | C10 H12 N2 O8 | 0.09789 | -3.3527 | 0.000228 | 1.541371 |
| 7-Methylguanosine | C11 H17 N5 O5 | 0.107662 | -3.21542 | 0.0004 | 1.643335 |
| LPE 18:1 | C23 H46 N O7 P | 0.200466 | -2.31857 | 0.000403 | 1.121394 |
| 2-Aminoadipic acid | C6 H11 N O4 | 0.167383 | -2.57877 | 0.000539 | 1.19392 |
| Glycerol-3-phosphate | C3 H9 O6 P | 0.188278 | -2.40906 | 0.000762 | 1.308586 |
| Phenylacetaldehyde | C8 H8 O | 0.169231 | -2.56294 | 0.002649 | 1.177826 |
| Catechin | C15 H14 O6 | 0.033854 | -4.88452 | 0.004117 | 1.966484 |
| 21-Deoxycortisol | C21 H30 O4 | 0.158974 | -2.65314 | 0.007374 | 1.144212 |
| Prostaglandin B2 | C20 H30 O4 | 0.115545 | -3.11347 | 0.007435 | 1.334035 |
| 3,3-Dimethylglutaric acid | C7 H12 O4 | 0.036215 | -4.78726 | 0.008097 | 1.723397 |
| Pinoresinol | C20 H22 O6 | 0.043465 | -4.52401 | 0.008272 | 1.842275 |
| N-[3-(aminosulfonyl)phenyl]-2,3-dihydro-1,4-benzodioxine-2-carboxamide | C15 H14 N2 O5 S | 0.058444 | -4.09681 | 0.008763 | 1.679405 |

**Table S4. The oligonucleotides used in this study.**

| Gene | Primer | Sequence(5′ to 3′) |
| --- | --- | --- |
| *TNF-α* | sense | 5′- CCCTCACACTCAGATCATCTTCT-3′ |
|  | antisense | 5′- GCTACGACGTGGGCTACAG-3′ |
| *IL-1β* | sense | 5′- GCAACTGTTCCTGAACTCAACT-3′ |
|  | antisense | 5′-ATCTTTTGGGGTCCGTCAACT-3′ |
| *IL-6* | sense | 5′- TAGTCCTTCCTACCCCAATTTCC-3′ |
|  | antisense | 5′-TTGGTCCTTAGCCACTCCTTC-3′ |
| *CCL2* | sense | 5′-TTAAAAACCTGGATCGGAACCAA-3′ |
|  | antisense | 5′-GCATTAGCTTCAGATTTACGGGT-3′ |
| *CCL3* | sense | 5′-ACCATGACACTCTGCAACCA-3′ |
|  | antisense | 5′-GTGGAATCTTCCGGCTGTAG-3′ |
| *CXCL1* | sense | 5′-CCACACTCAAGAATGGTCGC-3′ |
|  | antisense | 5′-TCTCCGTTACTTGGGGACAC-3′ |
| *Vegf* | sense | 5′-TTACTGCTGTACCTCCACC-3′ |
|  | antisense | 5′-ACAGGACGGCTTGAAGATG-3 |
| *NOS2* | sense | 5′-GTTCTCAGCCCAACAATACAAGA-3′ |
|  | antisense | 5′-GTGGACGGGTCGATGTCAC-3 |
| *ZO-1* | sense | 5′-GCCGCTAAGAGCACAGCAA-3′ |
|  | antisense | 5′-TCCCCACTCTGAAAATGAGGA-3 |
| *Occludin* | sense | 5′-CCCAGGCTTCTGGATCTATGT-3′ |
|  | antisense | 5′-TCCATCTTTCTTCGGGTTTTCA-3 |
| *Claudin-3* | sense | 5′-ACCAACTGCGTACAAGACGAG-3′ |
|  | antisense | 5′-CAGAGCCGCCAACAGGAAA-3 |
| *ler* | sense | 5′-CGACCAGGTCTGCCC-3′ |
|  | antisense | 5′-GCGCGGAACTCATC-3 |
| *tir* | sense | 5′-CCATGGAGAGCAGACGTAGCT-3′ |
|  | antisense | 5′-CGGTGATCCTGGATTTAACCTT-3 |
| *rpoA* | sense | 5′-GCGCTCATCTTCTTCCGAAT-3′ |
|  | antisense | 5′-CGCGGTCGTGGTTATGTG-3′ |
| *Csn1* | sense | 5′-CCTTTCCCCTTTGGGCTTAC-3′ |
|  | antisense | 5′-TGAGGTGGATGGAGAATGGA-3′ |
| *Csn2* | sense | 5′-CTTCAGAAGGTGAATCTCATGGG-3′ |
|  | antisense | 5′-CAGATTAGCAAGACTGGCAAGG-3′ |
| *Csn3* | sense | 5′-TCGACCCCATTACTCCCATTGTGT-3′ |
|  | antisense | 5′-TGTAAAAGGTAAGGGAAGACGAGAAAGAT-3′ |
| *Wap* | sense | 5′-AACATTGGTGTTCCGAAAGC-3′ |
|  | antisense | 5′-AGGGTTATCACTGGCACTGG-3′ |
| *GAPDH* | sense | 5′-AACTTTGGCATTGTGGAAGG-3′ |
|  | antisense | 5′-ACACATTGGGGGTAGGAACA-3′ |
